# Phytochrome interacting factor 3 regulates pollen mitotic division through auxin signalling and sugar metabolism pathways in tomato

**DOI:** 10.1111/nph.17878

**Published:** 2021-12-09

**Authors:** Dandan Yang, Yue Liu, Muhammad Ali, Lei Ye, Changtian Pan, Mengzhuo Li, Xiaolin Zhao, Fangjie Yu, Xinai Zhao, Gang Lu

**Affiliations:** ^1^ Department of Horticulture Zhejiang University Hangzhou 310058 China; ^2^ Department of Stem Cell Biology Centre for Organismal Studies Heidelberg University Im Neuenheimer Feld 230 Heidelberg 69120 Germany; ^3^ Key Laboratory of Horticultural Plant Growth, Development and Quality Improvement Ministry of Agricultural Zhejiang University Hangzhou 310058 China

**Keywords:** auxin, mitosis, phytochrome interacting factor, pollen development, *Solanum lycopersicum* (tomato), sugar

## Abstract

- The development of viable pollen determines male fertility, and is crucial for reproduction in flowering plants. Phytochrome interacting factor 3 (PIF3) acts as a central regulator of plant growth and development, but its relationship with pollen development has not been determined.
- Through genetic, histological and transcriptomic analyses, we identified an essential role for SlPIF3 in regulating tomato (*Solanum lycopersicum*) pollen development. Knocking out *SlPIF3* using clustered regularly interspaced short palindromic repeats/CRISPR‐associated protein 9 resulted in pollen mitosis I arrest, and a failure to form viable pollen. We further demonstrated that both glutamate synthase 1 (*SlGLT1*) and cell wall invertase 9 (*SlCWIN9*), involved in auxin and sugar homeostasis, respectively, colocalised with *SlPIF3* in the anthers and were directly regulated by SlPIF3. Knockout of either *SlGLT1* or *SlCWIN9* phenocopied the pollen phenotype of *SlPIF3* knockout (*Slpif3*) lines.
- *Slpif3* fertility was partially restored by exogenous auxin indole‐3‐acetic acid in a dose‐dependent manner.
- This study reveals a mechanism by which *SlPIF3* regulates pollen development and highlights a new strategy for creating hormone‐regulated genic male sterile lines for tomato hybrid seed production.

## Introduction

In flowering plants, the development of mature pollen in the anthers is critical for fertility and genetic diversity, and failure of this process leads to male sterility (Chen & Liu, 2014). Mature pollen development consists of meiotic and mitotic divisions: after meiotic division microspores are generated by microsporocytes and each microspore undergoes asymmetric mitotic division, referred to as pollen mitosis I (PMI), forming the vegetative cell and the generative cell. Finally, the generative cell divides into two sperm cells during pollen mitosis II (PMII), occurring within the anther (as in *Arabidopsis thaliana* and rice) or pollen tube (as in tomato) (McCormick, 2004; Borg *et al*., 2009). Mitosis in pollen is tightly controlled by cyclin/cyclin‐dependent kinase (cyclin/CDK) complexes, whose activity is negatively regulated by CDK inhibitor (CKI) proteins (Huntley & Murray, 1999). It has been reported that mutations in cell cycle regulators in *A. thaliana* can impair mitosis progression and result in gametophytic lethality (Nowack *et al*., 2006; Liu & Qu, 2008; Takatsuka *et al*., 2015). However, the mechanisms underlying mitotic cell cycle progression during male gametophyte development are still largely unknown.

Successful pollen development is regulated by both extracellular and intracellular signals, including the phytohormone auxin (Chen *et al*., 2007; Cecchetti *et al*., 2008, 2017). Two auxin biosynthesis genes, *YUC2* and *YUC6*, are predominantly expressed in the *A. thaliana* anther procambium, endothecium, tapetum, tetrads and microspores, and *yuc2yuc6* double mutants cannot progress through PMI and therefore form nonviable pollen grains (Cheng *et al*., 2006; Cecchetti *et al*., 2008). Ectopic expression of the *YUC2* gene in microsporocytes or microspores, but not in the tapetum, can rescue pollen abortion in *yuc2yuc6*, suggesting that spatially coordinated auxin distribution is required for the early stages of pollen development (Yao *et al*., 2018). Recently, it has been reported that auxin mainly accumulated in the anther from the microspore stage to the bicellular pollen grain stage and mediates haploid microspore‐to‐male gametophyte transition in both *A. thaliana* and tomato (*Solanum lycopersicum*) (Chen *et al*., 2018; Yao *et al*., 2018), but it is not known whether or how auxin regulates mitotic progression in pollen.

Another factor that can influence pollen development is the level of carbohydrates in anthers, as carbohydrates are important nutrients for sustaining pollen development (Chen *et al*., 2007; Min *et al*., 2014). In this regard, an important factor in carbohydrate metabolism is the activity of invertase (INV) enzymes, which hydrolyse sucrose into glucose and fructose. INVs are located in either the apoplast, vacuole or cytosol, where they are referred to as CWINs, VINs and CINs, respectively (Wan *et al*., 2018). INVs, including *NtNin88* in tobacco (Goetz *et al*., 2001), *SlLIN5* in tomato (Zanor *et al*., 2009) and *GhVIN1* in cotton (Wang & Ruan, 2016), participate in anther and pollen development by modulating sugar homeostasis and signalling (Cho *et al*., 2006). However, the molecular mechanism underlying sugar‐regulated pollen development is unclear.

Genetic analyses have identified some transcription factors involved in pollen development and maturation in *Arabidopsis* and rice, including many basic helix–loop–helix (bHLH) family members (Sorensen *et al*., 2003; Li *et al*., 2006). Phytochrome interacting factors (PIFs), a subset of the bHLH family, regulate multiple aspects of growth and development, such as seed germination, thermophotomorphogenesis, high temperature‐induced early flowering and seedling freezing tolerance, by coordinating development with different signals including auxin and sugar signalling (Franklin *et al*., 2011; Leivar & Monte, 2014; Rosado *et al*., 2016). For example, *Arabidopsis* PIF4 is a central hub regulator involving auxin biosynthesis and signalling to promote hypocotyl and petiole elongation (Hornitschek *et al*., 2012); PIF5 acts as a master switch in sugar‐induced auxin biosynthesis in *A. thaliana* seedlings (Sairanen *et al*., 2013). Recently, our group demonstrated that SlPIF4 plays an essential role in regulating the adaptation of tomato pollen development to low temperature stress via anther tapetal cell death (Pan *et al*., 2021). According to our previously published transcriptome data, *SlPIF3* and *SlPIF4* are highly expressed in tomato anthers (Chen *et al*., 2018; Pan *et al*., 2019). Given that *PIF3* and *PIF4* are known to co‐ordinately regulate plant development and responses to environmental stimuli, such as age‐triggered and dark‐induced leaf senescence (Song *et al*., 2014) and freezing tolerance (Jiang *et al*., 2020) in *A. thaliana*, we hypothesised that SlPIF3 might play important roles in the regulation of male development.

In this study, we demonstrated that SlPIF3 was directly involved in the regulation of pollen development in tomato. The *Slpif3* mutant was shown to exhibit a defective pollen phenotype with a poor germination rate caused by the arrest of PMI, accompanied by auxin deficit and lower soluble sugar levels in anthers compared with wild‐type plants. Chromatin immunoprecipitation (ChIP)‐seq and protein interaction assays revealed that SlPIF3 affects auxin biosynthesis and sugar metabolism in anthers by directly regulating the expression of *SlGLT1* and *SlCWIN9*. In summary, our study revealed that *SlPIF3* regulates pollen development, and suggested a novel strategy for the establishment of male sterile lines in tomato.

## Materials and Methods

### Plant materials and growth conditions

Tomato (*S*. *lycopersicum*) cultivar ‘Micro‐Tom’, provided by the Tomato Genetics Resource Center (University of California, Davis), was used for all gene‐transfer experiments and as the wild‐type. Tomato and *Nicotiana benthamiana* plants were grown in controlled growth chambers under 16 h : 8 h, light (300 μmol photons m^−2^ s^−1^) : dark, 25 ± 1°C : 20 ± 1°C cycles, with a relative humidity of 60–70%.

### Generation of transgenic plants

The *SlPIF3‐*knockout, *SlCWIN9*‐knockout and *SlGLT1*‐knockout mutants were generated using the clustered regularly interspaced short palindromic repeats/CRISPR‐associated protein 9 (CRISPR/Cas9) system (Pan *et al*., 2016) (Supporting Information Methods S1 for details). To develop *SlPIF3* overexpression lines, the full‐length coding sequence of SlPIF3 was cloned into the pFGC1008‐3HA vector (Yan *et al*., 2020), which harbours a CaMV 35S promoter. The vector was transformed into *Agrobacterium tumefaciens* strain GV3101 for transformation into tomato using the leaf‐disc method (Sun *et al*., 2006). The *DR5:GUS* transgenic line used for histochemical β‐glucuronidase (GUS) staining was generated in our previous study (Pan *et al*., 2019). The primers used are listed in Dataset S1.

### *In situ* hybridisation and subcellular localisation

*In situ* hybridisation was performed as previously described (Chen *et al*., 2018). Here, 295‐bp *SlPIF3*, 239‐bp *SlGLT1* and 275‐bp *SlCWIN9* coding sequence (CDS) fragments were each amplified and used as templates for transcription with SP6 and T7 RNA polymerases to produce digoxigenin‐labelled RNA probes (Roche, Germany). SlPIF3 subcellular localisation was performed as previously reported (Pan *et al*., 2021). The primers used are listed in Dataset S1.

### Phenotypic analysis

Pollen viability was determined by Alexander and fluorescein diacetate (FDA) staining (Alexander, 1969; Wang *et al*., 2008). 4′,6‐Diamidino‐2‐phenylindole (DAPI) staining was performed to analyse the meiotic and mitotic processes as previously described (Regan & Moffatt, 1990). DNA content was quantified and the DAPI fluorescence measured using ImageJ software and images taken with constant settings as previously described (Gusti *et al*., 2009). *In vitro* pollen germination was performed according to a previous report (Song *et al*., 1999), as was scanning electron microscopy (SEM) and transmission electron microscopy (TEM) (Chen *et al*., 2018). Terminal deoxynucleotidyl transferase (TdT)‐mediated dUTP nick end labelling (TUNEL) assays were performed according to the manufacturer’s instructions using a TUNEL apoptosis detection kit (Roche, Switzerland) with 10 μm anther paraffin‐embedded sections (Methods S2 for details).

### Transcriptome profiling and quantitative real‐time PCR analyses

Illumina sequencing libraries were constructed according to the manufacturer’s instructions (Illumina, San Diego, CA, USA) and then sequenced using an Illumina HiSeq 4000 system by Novogene Biotech (Novogene, Beijing, China). Quantitative real‐time polymerase chain reaction (qRT‐PCR) was performed using the SYBR® Green Real‐time PCR Master Mix (Toyobo, Osaka, Japan) on a Bio‐Rad CFX96 system (Bio‐Rad, USA). *SlUBI3* was used as an internal reference (Gutierrez *et al*., 2008; Fuentes *et al*., 2016; Pan *et al*., 2021). The relative genes expression levels were calculated using the $2^{‐\Delta \Delta C_{t}}$ method (Kenneth & Thomas, 2002) (Methods S3 for details). The primers used for qRT‐PCR analysis are listed in Dataset S1.

### Yeast‐one‐hybrid assays

Yeast‐one‐hybrid assays were carried out using the Matchmatch™ Gold Yeast One‐Hybrid System (Clontech, Mountain View, CA, USA). The promoter sequences containing a G‐box from the putative target genes were cloned into the pAbAi vector, and the full‐length *SlPIF3* cDNA was cloned into the pGADT7 vector. The pAbAi‐baits were transformed into the Y1HGold yeast genome and screened on selective drop out (SD)−Ura medium with different aureobasidin A (AbA) concentrations. The AD‐prey vectors were transformed into the bait strain and screened on an SD/−Leu/AbA plate. The primers used are listed in Dataset S1.

### ChIP‐seq and ChIP‐qPCR analysis

Here, *c*. 5 g of tomato anthers from *35S:SlPIF3‐3HA* transgenic plants, comprising a mixture of equal quantities at different developmental stages (stages II–IV) were harvested. The sample was fixed for 10 min under a vacuum at room temperature in 20 ml of 1% formaldehyde solution, followed by 5 min quenching with 125 mM glycine. Chromatin extracted from the samples was immunoprecipitated with anti‐HA antibody (Abcam, London, UK) and libraries were sequenced on an Illumina NovaSeq6000 system by Romics Biotech (Romics, Shanghai, China). ChIP‐qPCR was performed following the manufacturer’s instructions for the EpiQuikTM Plant ChIP Kit (Epigentek, Farmingdale, NY, USA). (Methods S4 for details). The primers listed in Dataset S1.

### Electrophoretic mobility shift assay

The pET‐32a‐His‐SlPIF3^1261–1605^ vector was generated using a truncated sequence of *SlPIF3* that encoded the bHLH domain. His‐tagged SlPIF3 protein was expressed in *Escherichia coli* strain Rosetta (DE3) and purified using a High Affinity Ni‐NTA Resin kit (GenScript, Nanjing, China). The putative target gene promoter fragments containing the G‐box and mutated G‐box region were synthesised as biotin‐labelled oligonucleotides according to a Biotin 3′ End DNA Labelling kit (Thermo Fisher Scientific, Waltham, MA, USA). Electrophoretic mobility shift assay (EMSA) was performed using a LightShift Chemiluminescent EMSA Kit (Thermo Fisher Scientific). The sequences are listed in Dataset S1.

### Exogenous indole‐3‐acetic acid treatment

Flower buds from *Slpif3‐6* plants were sprayed with 10^−5^, 10^−4^, or 10^−3^ M indole‐3‐acetic acid (IAA) (Phytotechlab, Shawnee Mission, KS, USA) with a 0.01% (v/v) aqueous solution of Silwet® L‐77. An aqueous solution of 0.01% (v/v) Silwet L‐77 was sprayed as a negative control. The flower buds were marked as buds at the microspore mother cell stage then solutions were sprayed onto all buds at 09:00 h with 2 d intervals for 6 d until the marked buds grew to the mature pollen stage. The pollen viability of the marked buds was determined to assess the auxin rescue efficiency.

### Measurement of endogenous IAA and soluble sugars levels

Measurement of endogenous IAA and soluble sugars levels was performed as previously reported (Chen *et al*., 2018) with minor modifications (Methods S5 for details).

### Assay of glutamate and glutamine contents

Measurement of free glutamate and glutamine was performed as previously reported (Man *et al*., 2011) with minor modifications (Methods S6 for details).

## Results

### The *Slpif3* mutant exhibits pollen abortion and almost complete male sterility

**Fig. 1:**
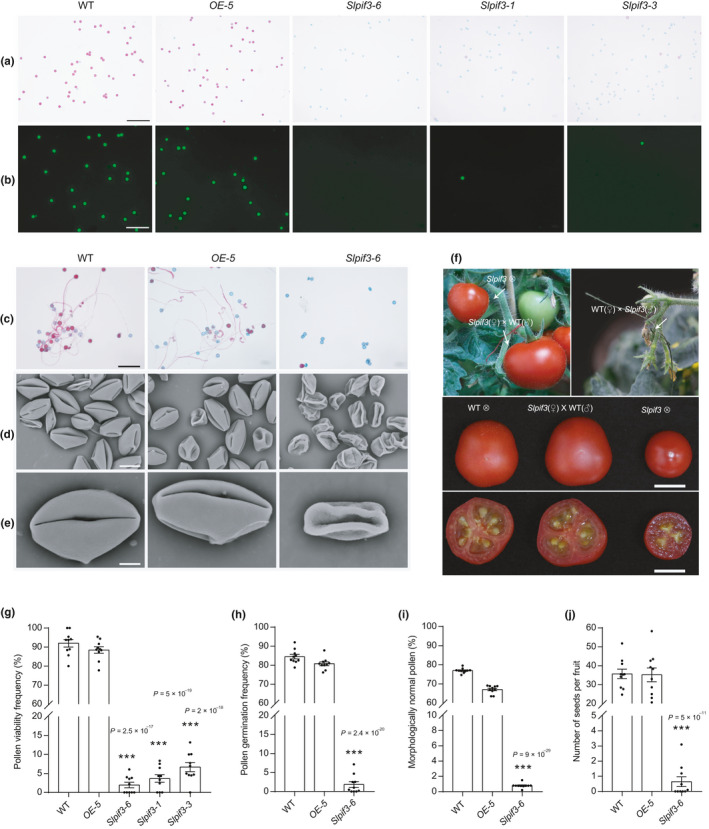
Knockout of *SlPIF3* leads to pollen abortion in tomato. Alexander (a) and fluorescein diacetate staining (b) of pollen grains from wild‐type (WT), *OE‐5*, *Slpif3‐6*, *Slpif3‐1* and *Slpif3‐3* mutants. (c) *In vitro* germination assay of pollen grain from the flower of WT, *OE‐5* and *Slpif3‐6*. (d, e) Scanning electron microscopy indicated that the *Slpif3‐6* pollen grain were shrinking compared with WT and *OE‐5*. (f) Reciprocal crossing of the *Slpif3‐6* mutant with WT plants. There is nearly no fruit set in *Slpif3‐6* self‐pollinated plants and the WT plants pollinated with *Slpif3‐6* pollen grains, but the *Slpif3‐6* flowers can develop normal fruits and seeds when they were pollinated with WT pollen grains. ♀, female parent; ♂, male parent; ⊗, self‐cross. Quantification of viable pollen (g), pollen germination (h), percentage of morphologically normal pollen (i) and number of seeds per fruit (j). (g–j) Individual values (dots) and means (bars) are shown. Each error bar represents the mean ± standard error (SE), *n* = 10 biologically independent samples. Each replicate included at least 200 pollen grains or 10 fruits. Asterisks indicate significant differences from WT control. *P‐*values in (g–j) were calculated using two‐tailed Student’s *t*‐test: ***, *P* < 0.001. Bars: (a, b) 200 µm; (c) 200 µm; (d) 15 µm; (e) 5 µm; (f) 1 cm.

To explore the biological function of *SlPIF3* during tomato pollen development, eight independent T0 *Slpif3* mutant lines were generated using the CRISPR/Cas9 system with guide RNA targeting regions upstream of the bHLH DNA‐binding domain. Putative off‐target site analysis indicated that mutagenesis of *SlPIF3* induced by the CRISPR/Cas9 system was specific and reliable (Fig. S1a). After sequencing targeted genomic regions of T2 populations derived from T1 mutants, we selected three homozygous/biallelic T2 mutant lines without Cas9‐protein for phenotypic observation. Among these mutants, *Slpif3‐1* and *Slpif3‐6* had a thymine (T) insertion and an adenine (A) insertion, respectively, resulting in a frame shift mutation and an early translation termination. Another biallelic mutant, *Slpif3‐3*, had a thymine (T) deletion and a large deletion (Fig. S1b). In addition, three homozygous overexpressing T2 lines (OE‐1, OE‐4 and OE‐5) were selected from 11 independent transgenic T0 lines as showing a significant increase in *SlPIF3* mRNA levels (Fig. S1c). None of the *SlPIF3* knockout or overexpressing plants displayed significant phenotypic differences from the wild‐type plants at the vegetative stage (Fig. S2a), including plant height and leaf size (Fig. S2b). Additionally, the floral organs of the transgenic plants were morphologically similar to those of the wild‐type (Fig. S2c,d). However, based on Alexander and FDA staining, the pollen viability was far lower in the homozygous *Slpif3‐6*, *Slpif3‐1* and *Slpif3‐3* lines (1.9%, 3.6% and 6.6%, respectively), than in the OE‐5 (88.5%) line and wild‐type (92.0%) (Fig. 1a,b,g). In addition, only 1.8% of the pollen grains from the *Slpif3‐6* germinated under *in vitro* culture conditions, while the germination rates were 80.8% and 84.5% for the OE‐5 line and wild‐type, respectively (Fig. 1c,h). In addition, SEM showed that 99.2% of the *Slpif3‐6* pollen grains appeared abnormally shrivelled and severely collapsed (Fig. 1d,e,i). Consistent with the disrupted pollen phenotype, knocking out *SlPIF3* resulted in an reduction in fruit size, weight and seed number (Figs 1f,j, S2e).

To further assess the effect of the mutation in *SlPIF3* on male and female development, reciprocal crosses were made between wild‐type and *Slpif3‐6* plants. In contrast with the *Slpif3‐6*, which developed only very small numbers of fruit with undeveloped seeds, pollination of *Slpif3‐6* ovaries with wild‐type pollen grains resulted in normal fruit and seed development, similar to that of self‐pollinated wild‐type plants, indicating that knocking out *SlPIF3* did not affect maternal fertility (Fig. 1f). However, pollination of wild‐type ovaries with *Slpif3‐6* pollen grains led to no fruit set (Fig. 1f). Therefore, knocking out *SlPIF3* impaired the function of male gametophytes but did not significantly affect female fertility.

### *SlPIF3* preferentially accumulates in pollen and fruit and is localised in nuclei

**Fig. 2:**
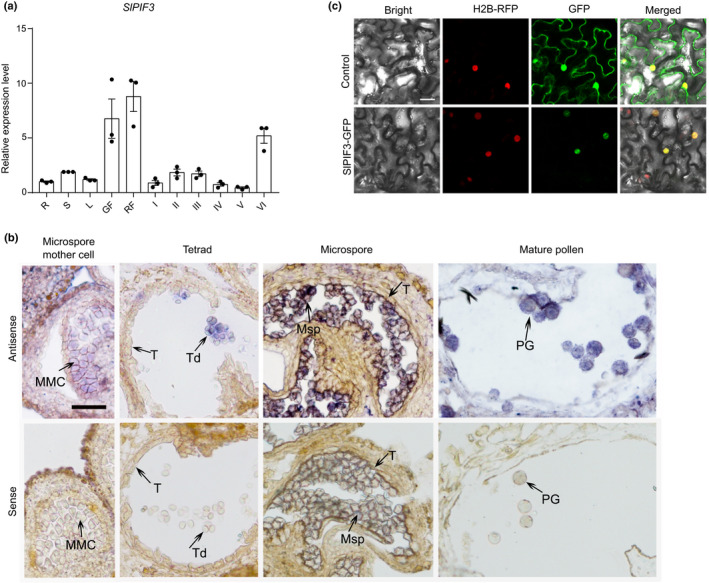
Expression profiles of *SlPIF3* in tomato. (a) Quantitative polymerase chain reaction (qPCR) analysis of the *SlPIF3* gene in various tissues of wild‐type (WT) plant. R, root; S, stem; L; leaf; GF, green fruit; RF, red fruit; I, anthers at pollen mother cell stage; II, anthers at tetrad stage; III, anthers at microspore stage; IV, anthers at polarised microspore stage; V, anthers at bicellular pollen stage; VI, anthers at mature pollen stage. The levels of gene expression normalised to *Ubiquitin* expression are shown relative to the root level set to 1. Individual values (dots) and means (bars) of three independent biological replicates are shown, each error bar represents the mean ± standard deviation. (b) RNA *in situ* result shows that the mRNA accumulation of *SlPIF3* in WT anthers. The signals of the antisense probe of *SlPIF3* were detected in microsporocytes, microspores and mature pollen grains. MMC, microspore mother cell; Msp, microspore; PG, pollen grain; T, tapetum; Td, tetrad. (c) Subcellular localisation of SlPIF3‐GFP in *Nicotiana benthamiana*. The pFGC‐GFP alone was used as control. GFP, green fluorescent protein fluorescence; H2B‐RFP, red fluorescent protein fluorescence of nucleic marker; Merge, merged image of GFP, H2B‐RFP and bright field signals. Representative images in (b, c) are from one of three independent experiments. The similar signals were detected in each experiment. Bars: (b) 50 µm; (c) 25 µm.

We next investigated *SlPIF3* expression during different stages of anther development using qRT‐PCR. *SlPIF3* transcription was detected in all the examined tissues, but expression was higher in developing reproductive organs, especially in fruit and anthers at the mature pollen stage (Fig. 2a). During anther development, *SlPIF3* exhibited low expression at stage I (microsporocyte) and slightly increased levels at stage II (tetrad) and stage III (microspore). However, transcript abundance significantly increased at stage VI (mature pollen). To further characterise the distribution patterns of *SlPIF3* in anthers, RNA *in situ* hybridisation analysis was performed. Signals from an *SlPIF3* antisense probe were detected in anthers, from the microspore mother cell stage to the mature pollen stage, and mainly in microsporocytes, microspores and pollen grains but not in the tapetum cells (Fig. 2b). These observations suggested that SlPIF3 is primarily involved in male reproductive development. Additionally, subcellular localisation analysis via heterologous expression of a SlPIF3‐GFP fusion protein in *N*. *benthamiana* leaf epidermal cells showed a fluorescence signal exclusively located in the cell nucleus (Fig. 2c), indicating that SlPIF3 is a nucleus‐localised protein.

### *SlPIF3* mutation disrupts pollen development before the bicellular pollen stage

**Fig. 3:**
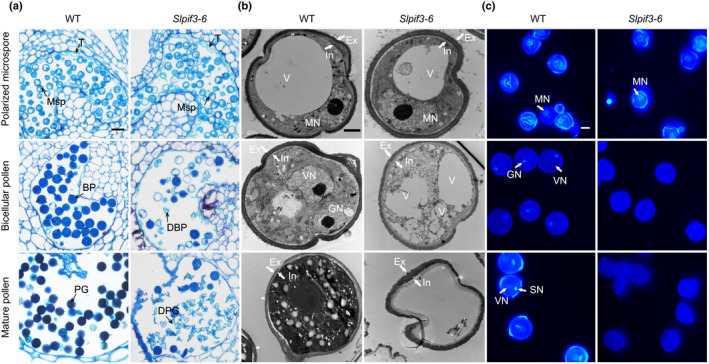
Knockout of *SlPIF3* disrupts tomato pollen development during the microspore to bicellular pollen transition stage. (a) Semithin cross‐sections of wild‐type (WT) and *Slpif3‐6* anthers from polarised microspore to mature pollen stage. The *Slpif3‐6* pollen development arrest before bicellular pollen and the mature *Slpif3‐6* pollen grains are collapsed. BP, bicellular pollen; DBP, degenerated bicellular pollen; DPG, degenerated pollen grain; Msp, microspore; PG, pollen grain; T, tapetum. Transmission electron microscopy (b) and 4′,6‐diamidino‐2‐phenylindole (DAPI) staining (c) of microspore and pollen grains of WT and *Slpif3‐6*. The nuclei were clearly present in the WT pollen but disappeared in *Slpif3‐6* in the bicellular pollen. Ex, exine; GN, generative nucleus; In, intine; MN, microspore nucleus; SN, sperm nucleus; V, vacuole; VN, vegetative nucleus. The experiments in (a**–**c) were repeated three times independently with consistent results. Bars: (a) 25 µm; (b) 2 µm; (c) 20 µm.

To characterise the pollen developmental defect resulting from a mutation in *SlPIF3*, we looked for morphological differences in transverse sections of anthers from wild‐type and *Slpif3‐6*. No differences were observed before the polarised microspore stage. *Slpif3‐6* showed normal meiosis, released microspores and even developed vacuolated microspores (Fig. S3a). However, at the bicellular pollen stage, *Slpif3‐6* pollen grains had an irregular appearance and degraded cytoplasm. At the mature pollen stage, the wild‐type anther locule was filled with pollen grains with dense cytoplasm, whereas the cytoplasm of *Slpif3‐6* pollen grains was further degenerated and shrunken (Fig. 3a). Indeed, anther tapetum development and degeneration in *Slpif3‐6* was the same as wild‐type, based on a TUNEL assay (Fig. S3c).

Consistently, TEM observation showed there were no obvious difference between *Slpif3‐6* and wild‐type pollen development before the polarised microspore stage, at which time a large vacuole and a distinct nucleus were observed (Fig. 3b). At the bicellular pollen stage, the wild‐type microspores underwent the first mitotic division (PMI) to form two nuclei and had a dense cytoplasm (Fig. 3b). However, in *Slpif3‐6*, the nuclei disappeared and, instead, many large vacuoles appeared. At the mature pollen stage, the cytoplasmic content of *Slpif3‐6* pollen grains had completely degenerated. However, the exine structure of the pollen grains remained intact, indicating that the integrity of the pollen wall was not significantly perturbed in *Slpif3‐6* (Figs 3b, S3b). Consistent with this finding, DAPI staining revealed that *Slpif3‐6* had one nucleus at the polarised microspore stage, similar to wild‐type plants; however, notable differences were apparent at the bicellular pollen stage (Fig. 3c), when *c*. 90% of the microspores in wild‐type plants had two nuclei, including a small generative cell nucleus with an intense fluorescence signal and a large vegetative cell nucleus with a faint signal. By contrast, only 1% of the *Slpif3‐6* microspores underwent PMI to arrive at the bicellular pollen stage (Fig. 3c). DAPI staining showed that the DNA content in the microspores at prophase of PMI was comparable between wild‐type and *Slpif3‐6* (Fig. S4a,b), suggesting that DNA replication occurred in the mutant, but is likely to be arrested at the S/G_2_ or M phase during mitotic cell cycle progression.

In summary, these results indicated that the male sterility of *Slpif3‐6* resulted from failure of PMI, which caused abnormal phenotypes in microspores, but not from defects in the tapetum and pollen cell wall development.

### *SlPIF3* regulates the expression of genes related to auxin signalling, sugar metabolism and the cell cycle

**Fig. 4:**
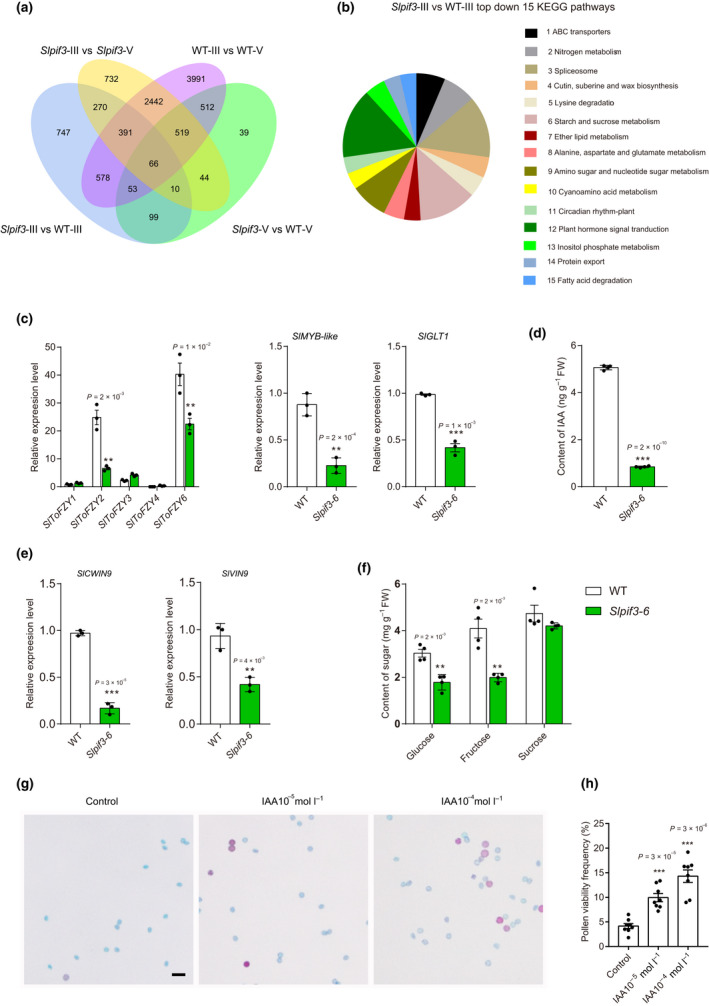
Comparison on auxin signalling and sugar signalling pathway in wild‐type (WT) and *Slpif3‐6* tomato anthers. Venn diagram (a) and KEGG analysis (b) of differentially expressed genes in WT and *Slpif3‐6* anthers. III, at microspore stage; V, at bicellular pollen stage. (c, e) Quantitative polymerase chain reaction (qPCR) analysis of the expression levels of auxin‐related and sugar‐related genes. The levels of gene expression normalised to *Ubiquitin* expression are shown relative to the WT level set to 1. Individual values (dots) and means (bars) are shown with three independent biological replicates, each error bar represents the mean ± standard deviation. Asterisks indicate significant differences between *Slpif3‐6* and WT plants. (d) Indole‐3‐acetic acid (IAA) levels in anthers of *Slpif3‐6* were diminished in comparison to WT. Individual values (dots) and means (bars) are shown with four independent biological replicates, each error bar represents the mean ± SD. Asterisks indicate significant differences between *Slpif3‐6* and WT plants. (f) Fructose, glucose and sucrose content in WT and *Slpif3‐6* anthers. Individual values (dots) and means (bars) are shown with four independent biological replicates, each error bar represents the mean ± SD. Asterisks indicate significant differences between *Slpif3‐6* and WT plants. (g) Alexander staining of *Slpif3‐6* pollen grains treated with 0, 10^–5^ or 10^–4^ mol l^–1^ IAA, respectively. (h) Percentage of viable pollen by Alexander staining. Individual values (dots) and means (bars) are shown. Each error bar represents the mean ± SE, *n* = 8 biologically independent samples. Each replicate included at least 200 pollen grains. Asterisks indicate significant differences between *Slpif3‐6* and WT plants. *P‐*values in (c–f, h) were calculated using two‐tailed Student’s *t*‐test: **, *P* < 0.01; ***, *P* < 0.001. Bar, (g) 50 µm.

To further elucidate the role of *SlPIF3* in pollen development, we compared the *Slpif3‐6* and wild‐type anther transcriptomes at the microspore (stage III) and bicellular pollen (stage V) stages (Table S1). An expression profile comparison indicated high reproducibility of the data sets (Fig. S5a,b). Compared with the wild‐type, in total, 2214 and 1342 differentially expressed genes (DEGs; log_2_|fold change| ≥ 1 and adjusted *P*‐value ≤ 0.05) were detected in *Slpif3‐6* anthers at stage III and stage V, respectively (Fig. 4a; Dataset S2). Among the DEGs, 1295 (58%) were expressed at lower levels in the mutant and 919 (42%) at higher levels at stage III, while these respective values were 905 (67%) and 437 (33%) at stage V. Only 228 DEGs between the wild‐type and *Slpif3‐6* were common at both stages. As *Slpif3‐6* pollen development arrest occurred before the bicellular pollen stage, we deduced that DEGs at the microspore stage (stage III) were more likely to be relevant to pollen abortion. Kyoto Encyclopedia of Genes and Genomes (KEGG) enrichment pathway analysis showed that the downregulated genes in mutant anthers at stage III were highly enriched in pathways such as ‘hormone signal transduction’, ‘starch and sucrose metabolism’ and ‘spliceosome’ (Fig. 4b; Dataset S3).

With regards to the hormone signal transduction pathways, many auxin‐related genes were differentially expressed between *Slpif3‐6* and wild‐type (Table 1). These included auxin influx carrier 1 (*AUX1*), transport inhibitor response 1 (*TIR1*), auxin response factor (*ARF*) genes and Gretchen Hagen 3 (*GH3*) family genes, all of which were expressed at substantially lower levels in *Slpif3‐6*. To investigate whether *SlPIF3* regulates pollen development through the auxin signalling pathway, we first examined auxin distribution during tomato anther development using DR5‐GUS transgenic plants (Ulmasov *et al*., 1997; Pan *et al*., 2019). GUS staining revealed that auxin mainly accumulated in anthers from the microspore to bicellular pollen stages (stages III–V) (Fig. S4c), consistent with previous reports that auxin was mainly detected from stages 10 (end of meiosis) to 12 (bicellular pollen) in *A. thaliana* anthers (Cecchetti *et al*., 2008, 2017). Anther cross‐sections showed intense blue staining in the microspore, tapetum and anther cell wall at stage III (Fig. S4d). We next compared the changes in expression of auxin biosynthesis genes in wild‐type and *Slpif3‐6* anthers at stage III. The mRNA levels of the key auxin biosynthesis genes *SlToFZY2* and *SlToFZY6* in *Slpif3‐6* anthers were 27% and 56%, respectively, of those in wild‐type anthers (Fig. 4c). In tomato, *ToFZYs* encoding flavin monooxygenases function as rate‐limiting enzymes in auxin biosynthesis (Expósito‐Rodríguez *et al*., 2011). Additionally, the *GLT1* gene encodes an NADH‐dependent glutamine‐2‐oxoglutarate aminotransferase (NADH‐GOGAT), which is a key enzyme involved in nitrogen assimilation and catalyses the conversion of glutamine to glutamate. Glutamine provides amino groups for the synthesis of anthranilate from chorismite, which is the first step in the tryptophan‐dependent auxin biosynthesis pathway (Cho *et al*., 2000; Man *et al*., 2011). In stage III, *SlGLT1* was expressed at significantly lower levels in *Slpif3‐6* than in wild‐type (Fig. 4c). In addition, the expression of *SlMYB‐like* gene, whose homologue in *A. thaliana* was previously shown to be involved in auxin biosynthesis (Rawat *et al*., 2009), was significantly lower in *Slpif3‐6* (Fig. 4c). In addition, *SlMYB‐like* was expressed at significantly higher levels in *SlPIF3* overexpression line anthers at stage III (Fig. S6a). As expected, the endogenous auxin content in *Slpif3‐6* was also lower than in wild‐type: levels were 17% of those in wild‐type anthers at stage III (Fig. 4d). Given the importance of PIFs in regulating plant photomorphogenesis through the brassinosteroid (BR) signal transduction pathway (Leivar & Monte, 2014) and the fact that the dwarf phenotype of ‘Micro‐Tom’ is based on a BR‐related mutation (Martí *et al*., 2006), we investigated whether BR participates in PIF3‐mediated pollen development. RNA‐seq data showed that the expression levels of both BR biosynthesis‐ and BR signalling‐related genes were not significantly different in *Slpif3‐6* compared with those of wild‐type (Fig. S5c), indicating that knocking out *SlPIF3* may not affect BR signalling during anther development. Notably, previous studies of both *A. thaliana* and tomato have shown that BR biosynthetic and signalling mutants have reduced pollen viability resulting from delayed tapetal degeneration (Ye *et al*., 2010; Yan *et al*., 2020), which is different from the phenotype of the *Slpif3‐6* (Fig. S3c).

**Table 1:** Differentially expressed genes between *Slpif3‐6* and wild‐type (WT) (*Slpif3‐6* vs WT) in tomato anther at the microspore stage.

Functional category	Gene name	Gene locus	Log_2_FC	*P*‐value
Auxin related	*SlLAX1*	Solyc09g014380.3	−0.75	1.40E−06
	*SlTIR1*	Solyc06g008807.1	−1.45	3.65E−09
	*SlIAA13*	Solyc09g090910.2	−0.74	0.00265
	*SlAFR‐like*	Solyc04g081235.1	−1.29	0.011121
	*SlARF9a*	Solyc08g082630.3	−0.66	4.90E−09
	*SlARF3*	Solyc02g077560.3	−0.64	9.23E−08
	*SlGH3.8*	Solyc02g064830.3	−2.20	1.23E−07
	*SlGH3.6*	Solyc07g053030.3	−2.23	4.79E−05
	*SlGH3.10*	Solyc02g092820.3	−1.53	1.62E−22
	*SlGLT1‐like*	Solyc08g044270.3	−3.94	1.18E−05
	*SlGLT1*	Solyc03g083440.3	−2.65	5.21E−34
	*SlGLS1*	Solyc03g063560.3	−1.01	2.04E−13
	*SlGLN1*	Solyc05g051250.3	−1.08	1.07E−06
	*SlGLN1‐2*	Solyc04g014510.3	−1.53	1.83E−06
Sugar related	*SlVIN9*	Solyc08g079080.3	−1.34	0.000578
	*SlCWIN9*	Solyc10g085650.2	−1.20	0.000421
	*SlBGLU17*	Solyc10g045240.2	−1.43	6.55E−18
	*SlBGLU46*	Solyc07g063390.3	−2.43	7.01E−24
	*SlBGLU3*	Solyc11g071650.2	−1.31	1.94E−11
	*SlBGLU40*	Solyc01g010390.3	−1.58	0.005197
	*SlSPS4*	Solyc11g045110.2	−1.27	0.000438
Kinesin related	*SlKinesin 7.4*	Solyc01g110190.3	−1.433	8.44E−09
	*SlKinesin1*	Solyc04g076310.3	−1.5947	0.000179
	*SlKinesin‐like*	Solyc02g084390.3	−1.4388	1.06E−17
	*SlKinesin2*	Solyc04g078610.3	−2.4585	7.02E−14
	*SlKIP1*	Solyc09g065550.3	−1.9257	5.50E−19
	*SlKinesin‐5*	Solyc08g081120.3	−1.7919	1.96E−23
	*SlKinesin3*	Solyc06g009780.3	−1.788	2.36E−15
	*SlKinesin4*	Solyc08g079710.3	−1.1017	5.88E−11
	*SlKinesin 7.2*	Solyc10g054080.2	−1.0782	6.78E−06
	*SlKinesin 7.3*	Solyc04g040110.3	−1.0368	8.16E−10
Myosin related	*SlMyosin XI*	Solyc07g041150.3	−2.6494	3.49E−26
	*SlMyosin XI‐F2*	Solyc01g081545.1	−2.234	1.54E−09
	*SlMyosin XI‐F*	Solyc01g081540.3	−1.3795	9.94E−13
	*SlMyosin XI‐E*	Solyc06g083960.2	−1.7781	1.69E−09
	*SlMyosin XI‐E2*	Solyc06g008530.2	−1.19	0.003903
	*SlMyosin XI‐I*	Solyc08g061500.2	−1.05	3.76E−10
	*SlMyosin XI‐A*	Solyc09g091080.3	−2.32	9.53E−06
Cyclin related	*SlCYCD2‐1*	Solyc12g087900.2	−2.66	0.000664
	*SlCYCE*	Solyc07g049350.3	−1.22	2.36E−09
	*SlCYCA‐like*	Solyc03g115510.2	−1.22	0.00017
	*SlKRP2*	Solyc09g061280.3	1.22	1.41E−22

The majority of carbon metabolism and sugar signalling genes, including INVs, beta‐glucosidases and sucrose synthetases, were expressed at lower levels in *Slpif3‐6* than in wild‐type at stage III (Table 1). For example, *SlCWIN9*, a cell wall INV gene, was highly expressed in wild‐type anthers but exhibited a 95% reduction in *Slpif3‐6* (Fig. 4e), and two‐fold higher expression in *SlPIF3* overexpressing plants (Fig. S6a). The mRNA levels of *SlVIN9* were also significantly lower in *Slpif3‐6* anthers (Fig. 4e). We observed that glucose levels were 43% lower and fructose levels were also significantly reduced at the microspore stage in *Slpif3‐6* anthers compared with the wild‐type. Surprisingly, no significant differences were observed in the sucrose level between *Slpif3‐6* and wild‐type plants (Fig. 4f), whereas the glucose, fructose and sucrose contents in the *SlPIF3* overexpressing line were similar to these of wild‐type (Fig. S6b).

At stage III, genes encoding kinesin and myosin, which are involved in spindle and cytoskeletal dynamics and organisation, were expressed at significantly lower levels in *Slpif3‐6* anthers compared with in wild‐type (Table 1). Moreover, the expression levels of many cell division‐related genes, such as *SlCYCA‐like* and *SlCYCD2;1*, were significantly decreased, whereas *SlKRP2* and *SlKRP4* expression levels were enhanced in *Slpif3‐6* anthers (Table 1; Fig. S6). While there was no significant difference between *SlKRP2*, *SlCYCD2;1* and *SlKinesin2* expression in *SlPIF3* overexpressing plants and the wild‐type (Fig. S6a), *SlKinesin1* and *SlKinesin3* expression was significantly increased in the overexpressing plants (Fig. S6a).

It is worth noting that the expression of genes involved in tapetum and pollen cell wall development showed no significant difference between *Slpif3‐6* and wild‐type plants (Table S2), which was consistent with the mutant phenotype.

### *Slpif3* mutant pollen viability can be partially rescued by exogenous auxin

To further assess the role of auxin in pollen development, *Slpif3‐6* flower buds were treated with different concentrations of exogenous auxin (10^–5^, 10^–4^ or 10^–3^ M IAA) and a 0.01% (v/v) aqueous solution of Silwet L‐77 was used as a mock control. IAA was sprayed onto the newly emerging flower buds three times at 2‐d intervals, allowing the microsporocytes to develop into mature pollen. The pollen viability of *Slpif3‐6* increased to 9.9% and 14.3% after exposure to 10^–5^ and 10^–4^ M IAA, respectively, whereas only 4.1% of pollen grains were viable after mock treatment, indicating that exogenous IAA treatment increased *Slpif3‐6* pollen viability (Fig. 4g,h), and that the rescue effect was increased with higher concentration of IAA. We observed that IAA concentrations ≥ 10^–3^ M caused abscission of many flower buds. Notably, an exogenous supply of glucose at different concentrations (0.1 and 0.01 M) did not markedly rescue pollen viability (data not shown).

To learn more about the partially rescued pollen viability, we monitored the change in expression of cell cycle‐related and sugar signalling genes in *Slpif3‐6* after treating with IAA (10^–4^ M). We found that *SlKRP2* and *SlKRP4* expression levels were significantly lower in *Slpif3‐6* after auxin treatment compared with the mock control (Fig. S6c), even *SlKRP2* expression reached wild‐type levels (Fig. S6c). By contrast, no significant difference in expression of *SlCWIN* genes was detected, whereas the expression levels of both *SlVIN2* and *SlVIN9* were higher in *Slpif3‐6* anthers after auxin treatment compared with the mock control (Fig. S6c). These results suggested that exogenous auxin treatment of *Slpif3‐6* altered the expression of cell cycle‐related and sugar‐related genes during anther development.

### SlPIF3 directly binds to *SlMYB‐like*, *SlGLT1* and *SlCWIN9* promoters

**Fig. 5:**
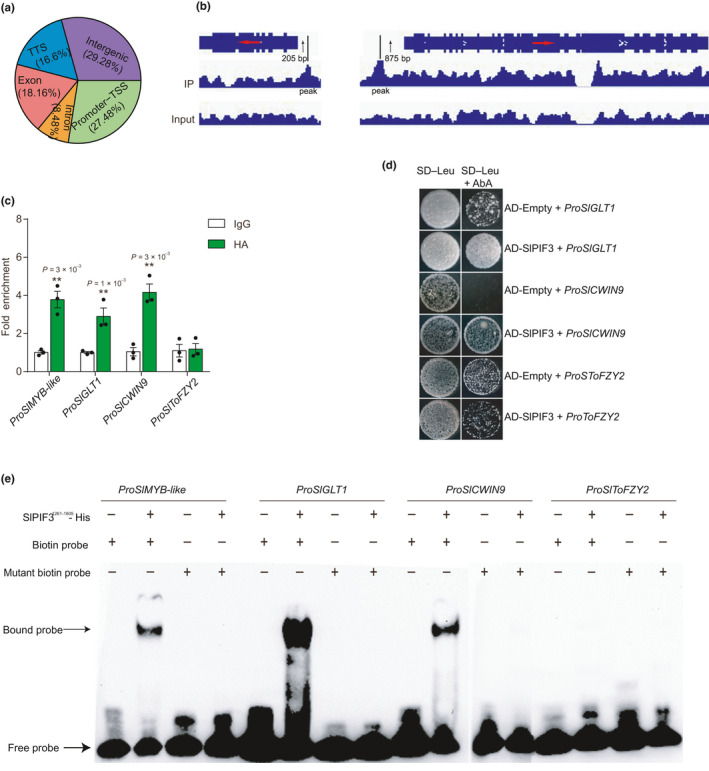
SlPIF3 directly binds to the promoters of *SlMYB‐like*, *SlGLT1* and *SlCWIN9*. (a) Distribution of SlPIF3 binding sites in the tomato genome. (b) ChIP‐seq shows that SlPIF3 binds to the promoter regions of *SlMYB‐like* and *SlGLT1* genes. Red arrows represent the direction of gene; black arrows indicate the length of peak away from ATG of gene. (c) Verification of SlPIF3 binding region by ChIP‐qPCR. DNA was recovered after the addition of anti‐HA and immunoglobulin G (IgG) antibodies. Antibody IgG was used as a template. Enrichment was confirmed by quantitative polymerase chain reaction (qPCR) using the primer sets (*ProSlMYB‐like*, *ProSlGLT1*, *ProSlCWIN9* and *ProSlToFZY2*) and the levels of gene expression normalised to *SlActin2* expression are shown relative to the IgG level set to 1. Individual values (dots) and means (bars) are shown with three independent biological replicates, each error bar represents the mean ± standard deviation. Asterisks indicate significant differences from IgG control. *P‐*values were calculated using two‐tailed Student’s *t*‐test: **, *P* < 0.01. (d) Yeast‐one‐hybrid analysis of SlPIF3 binding to the promoter region of *SlGLT1*, *SlCWIN9* and *SlToFZY2*. Interaction was determined on selective dropout (SD)/−Leu medium in the presence of aureobasidin A (AbA). (e) Electrophoretic mobility shift assay (EMSA) showing that SlPIF3 directly binds to the core sequence in a G‐box‐dependent manner. Biotin probe or mutant biotin probe for DNA oligomers with or without the G‐box, whichwere identified by the promoter region of the target genes labelled with biotin. Experiments (d, e) were repeated three times independently with consistent results.

To identify the direct target genes of SlPIF3 in anthers, we performed genome‐wide ChIP‐seq. In total, we identified 2442 high‐confidence peaks by comparing significant SlPIF3‐enriched peaks with the input control (Dataset S4). SlPIF3 bound to various genomic sites, a high percentage (70.2%) of which was subsequently assigned to genic bodies; moreover, the highest percentage (27.48%) was found in the region −3.0 kb from the transcription start site (TSS) (Fig. 5a). These data suggested that SlPIF3‐binding sites were strongly enriched in the promoter region, peaking *c*. 150 bp upstream of the TSS (Fig. S7a).

We then searched for significantly enriched motifs and found that the G‐box (CACGTG) was the most statistically overrepresented motif (Fig. S7b) (Ferré‐D’Amaré *et al*., 1993). Subsequently, by combining the results from ChIP‐seq and RNA‐seq, 147 and 75 SlPIF3‐targeting genes were identified as putative direct SlPIF3 binding targets for transcriptional regulation in anthers at stage III and stage V, respectively (Fig. S7c; Dataset S5).

Although *SlToFZY2* and *SlToFZY6* transcript levels were lower in *Slpif3‐6* compared with the wild‐type, the ChIP‐seq results did not suggest that SlPIF3 directly targeted these two genes. Based on the ChIP‐seq and RNA‐seq results, the putative target genes, *SlMYB‐like* and *SlGLT1*, were selected for further protein interaction analysis (Fig. 5b). ChIP‐qPCR revealed that *SlMYB‐like* and *SlGLT1* showed 3.8‐fold and 2.9‐fold binding enrichment, respectively, compared with the input control (Fig. 5c). However, no direct association of SlPIF3 with the promoter of *SlToFZY2* was found (Fig. 5c). Given that the expression of the sugar metabolism gene *SlCWIN9* was significantly lower in *Slpif3‐6* and that the *SlCWIN9* promoter region contained a G‐box sequence, we investigated whether SlPIF3 directly bound to *SlCWIN9*. ChIP‐qPCR data demonstrated that the *SlCWINV9* promoter fragment was enriched 4.2‐fold compared with the control (Fig. 5c). These findings indicated that SlPIF3 directly targets *SlMYB‐like*, *SlGLT1* and *SlCWIN9* during anther development. Furthermore, a yeast‐one‐hybrid (Y1H) assay demonstrated that SlPIF3 can directly interact with the promoters of *SlGLT1* and *SlCWIN9* (Fig. 5d). Finally, to confirm the interaction, EMSA was performed with a biotin‐labelled probe containing the G‐box sequence or its mutated version. We observed a mobility shift with the probe containing the G‐box element from the *SlMYB‐like*, *SlGLT1* and *SlCWIN9* promoter regions when incubated with a His‐SlPIF3 fusion protein, whereas no mobility shift was detected with the probe containing the mutated sequence from these regions (Fig. 5e). By contrast, the presence of the *SlToFZY2* promoter E‐box did not result in a mobility shift (Fig. 5e). This experiment indicated that SlPIF3 binds to the G‐box sequence in the *SlCWIN9*, *SlGLT1* and *SlMYB‐like* promoters.

### Knocking out *SlCWIN9* and *SlGLT1* induces pollen developmental defects

**Fig. 6:**
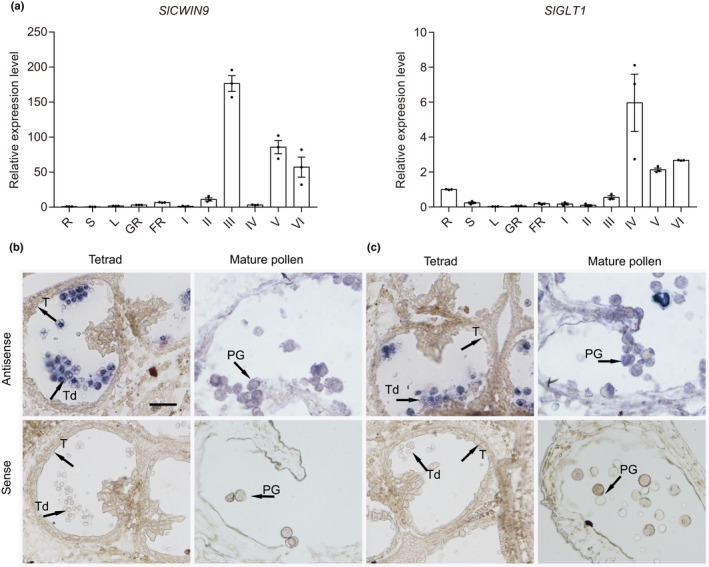
Expression profiles of *SlCWIN9* and *SlGLT1* in tomato. (a) Quantitative polymerase chain reaction (qPCR) analysis of *SlCWIN9* and *SlGLT1* genes in various tissues of wild‐type (WT) plants. R, root; S, stem; L; leaf; GF, green fruit; RF, red fruit; I, anthers at pollen mother cell stage; II, anthers at tetrad stage; III, anthers at microspore stage; IV, anthers at polarised microspore stage; V, anthers at bicellular pollen stage; VI, anthers at mature pollen stage. The levels of gene expression normalised to *Ubiquitin* expression are shown relative to the root level set to 1. Individual values (dots) and means (bars) of three independent biological replicates are shown, each error bar represents the mean ± standard deviation. (b, c) RNA *in situ* shows the mRNA accumulation of *SlCWIN9* (b) and *SlGLT1* (c) in WT anthers. The signals of the antisense probes of *SlCWIN9* and *SlGLT1* were detected in tetrads and pollen grains. PG, pollen grain; T, tapetum; Td, tetrad. The experiments in (b, c) were repeated three times independently with consistent results. Bar, (b) 50 µm.

**Fig. 7:**
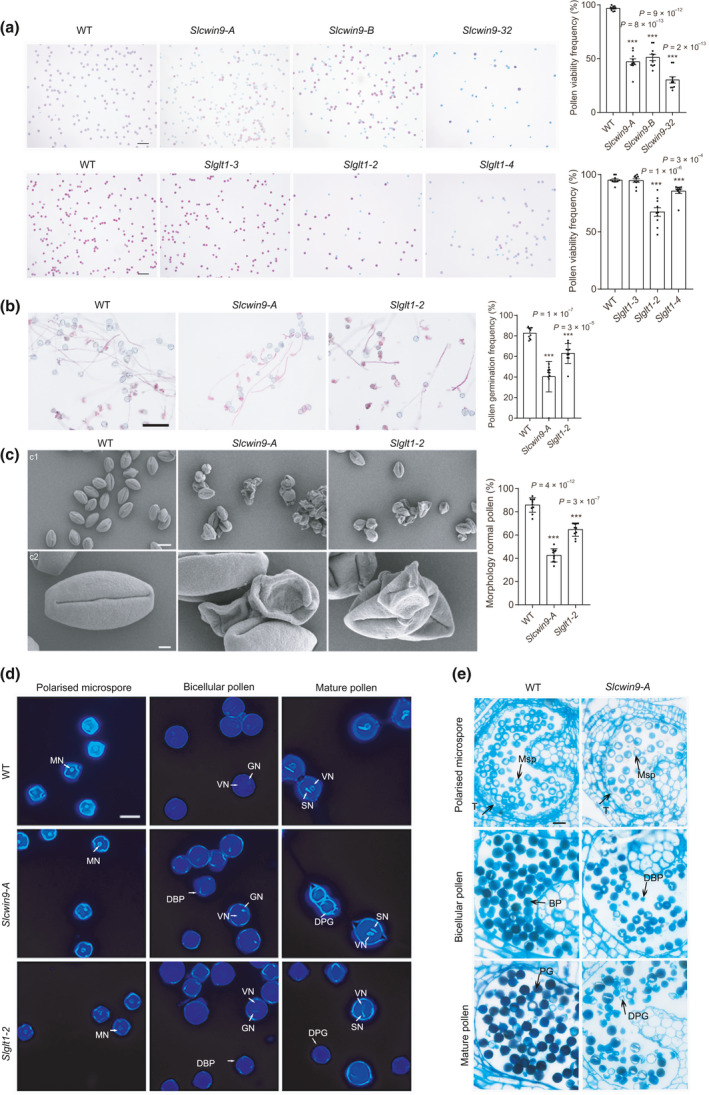
Knockout of *SlCWIN9* and *SlGLT1* induces pollen defects in tomato. (a) Pollen viability determined by Alexander staining in *Slcwin9* and *Slglt1* clustered regularly interspaced short palindromic repeats/CRISPR‐associated protein 9 (CRISPR/Cas9) knockout lines. Individual values (dots) and means (bars) are shown. (b) *In vitro* germination assay of pollen grain from the flower of WT (WT), *Slcwin9‐A* and *Slglt1‐2*. (c) Scanning electron microscopy of pollen grain from the flower of WT, *Slcwin9‐A* and *Slglt1‐2*. (d) 4′,6‐Diamidino‐2‐phenylindole (DAPI) staining of microspores and pollen grains of WT, *Slcwin9* and *Slglt1* in polarised microspore, bicellular pollen, and mature pollen stages. DBP, degenerated bicellular pollen; DPG, degenerated pollen grain; GN, generative nucleus; MN, nucleus of microspore; SN, sperm nucleus; VN, vegetative nucleus. (e) Semithin cross‐sections of WT and *Slcwin9‐A* anthers at polarised microspore, bicellular pollen and mature pollen stages. *Slcwin9‐A* pollen development arrest in bicellular and mature pollen grain is collapsed. BP, bicellular pollen; DBP, degenerated bicellular pollen; DPG, degenerated pollen grain; Msp, microspore; PG, pollen grain; T, tapetum. (a–c) Each error bar represents the mean ± standard error (SE), *n* = 10 biologically independent samples. Each replicate included at least 200 pollen grains. Asterisks indicate significant differences from the WT control. *P‐*values in (a–c) were calculated using two‐tailed Student’s *t*‐test: ***, *P* < 0.001. The experiments in (d, e) were repeated three times independently with similar results. Bars: (a, b) 100 µm; (c1) 20 μm; (c2) 3 μm; (d) 20 μm; (e) 25 µm.

To further investigate whether *SlCWIN9* and *SlGLT1* were involved in pollen development, their expression patterns and biological function were characterised. *SlCWIN9* mRNA accumulated preferentially in anthers, with the highest expression level at stage III (Fig. 6a). RNA *in situ* hybridisation analysis revealed that *SlCWIN9* was mainly expressed in the tetrads and mature pollen grains, and not expressed in the tapetal cells (Fig. 6b), which was also consistent with *SlPIF3* mRNA distribution in anthers (Fig. 2c). *SlCWIN9* CRISPR/Cas9 knockout lines were generated and three homozygous T2 mutant lines without T‐DNA components were selected (*Sl*cw*in9‐A*, *Slcwin9‐B* and *Sl*cw*in9‐32*). Both *Slcwin9‐A* and *Slcwin9‐B* had an adenine (A) insertion, whereas *Slcwin9‐32* had a 2‐bp deletion (Fig. S8a). The *Slcwin9* mutant displayed normal vegetative growth and flowering (Fig. S8b,c), whereas pollen viability was decreased to 47.2%, 51.2% and 30.3% in *Slcwin9‐A*, *Slcwin9‐B* and *Slcwin9‐32* lines, respectively, compared with 97.9% in wild‐type plants (Fig. 7a), suggesting that *SlCWIN9* contributes to male gametophyte development. Only 40.2% of *Slcwin9‐A* pollen grains germinated compared with 82.5% in wild‐type plants under *in vitro* culture conditions (Fig. 7b). SEM showed that 57.4% of the *Slcwin9‐A* pollen grains appeared extremely shrivelled (Fig. 7c), and DAPI staining revealed that 49% of the *Slcwin9‐A* mutant microspores failed to undergo PMI and arrested before the bicellular pollen stage (Fig. 7d). In addition, transverse semithin anther sections from wild‐type and *Slcwin9‐A* plants further showed that the pollen developmental defect was primarily observed after the polarised microspore stage, consistent with the *Slpif3‐6* mutant phenotype (Fig. 7e). As expected, both glucose and fructose levels were significantly lower in *Slcwin9‐A* (Fig. S9c). Notably, the expression level of *SlKRP2*, a CDK suppressor, was three‐fold higher in *Slcwin9‐A* anthers than in wild‐type anthers at stage III, whereas *SlCYCD2;1* and *SlKinesin1* levels were significantly lower (Fig. S9a). The change in expression levels of these cell cycle‐related genes induced by the *SlCWIN9* knockout was therefore quite similar to those in the *Slpif3‐6* mutant (Table 1).

*SlGLT1* mRNA also accumulated predominantly in tomato anthers, with the highest transcript level at stage IV (Fig. 6a). Consistent with *SlPIF3* expression, RNA *in situ* hybridisation revealed that the *SlGLT1* antisense signal was mainly present in the tetrads and mature pollen grains, and not in the tapetal cells (Fig. 6c). Three homozygous *SlGLT1* knockout T2 mutant lines without the CRISPR/Cas9 transgene were generated. These lines had a 3‐bp deletion (*Slglt1‐3*), an adenine (A) insertion (*Slglt1‐2*) and a 13‐bp deletion (*Slglt1‐4*) (Fig. S8a). No visible phenotypic differences were observed between the *Slglt1* mutant and wild‐type plants during the vegetative stage or in flower morphology (Fig. S8b,c). Compared with the wild‐type, the pollen viability of the *Slglt1‐2* and *Slglt1‐4* mutant lines was 67.4% and 85.5%, respectively, whereas 94.8% of wild‐type pollen grains were viable, as evidenced by staining with Alexander’s reagent (Fig. 7a), and *Slglt1‐3* with a 3‐bp deletion exhibited similar pollen viability to that of the wild‐type. *In vitro Slglt1‐2* pollen germination frequency was 24% lower than that of wild‐type pollen grains (Fig. 7b); SEM revealed that 35.32% of the mutant pollen had irregular shapes and a collapsed phenotype (Fig. 7c). DAPI staining of *Slglt1‐2* mutant pollen showed that 36% of the microspores failed to undergo first mitosis and developed bicellular pollen grains (Fig. 7d), consistent with the *Slpif3‐6* phenotype. To investigate whether *SlGLT1* affected anther glutamine and glutamate levels, we measured the expression levels of glutamine synthetases (GSs) by qRT‐PCR and found that *SlGS1;1*, *SlGS1;2* and *SlGS2* were all expressed at lower levels in the *Slglt1‐2* mutant anthers at microspore stage compared with the wild‐type (Fig. S9b). In addition, high‐performance liquid chromatography analysis showed that the contents of both glutamate and glutamine were significantly lower in *Slglt1‐2* than in wild‐type (Fig. S9d). To further explore the effects of changed glutamine and glutamate levels on auxin levels in *Slglt1‐2* anthers, the expression patterns of auxin‐related genes in the anthers were compared between *Slglt1‐2* and wild‐type. The transcript levels of three anthranilate synthase (AS) genes were significantly lower in *Sglt1‐2* than in wild‐type. Furthermore, several key genes involved in auxin biosynthesis, including *SlToFZY2* and *SlToFZY6*, were similarly lower (Fig. S9b). Consistent with this observation, the endogenous auxin concentration in *Slglt1‐2* anthers at stage III was lower compared with wild‐type (Fig. S9e).

## Discussion

The PIF family was originally identified as a phytochrome interacting protein and is known as a signal hub for multiple development processes. However, most of the studies have focused on PIF’s function in vegetative organs (Leivar & Monte, 2014), and it is largely unknown whether or how they are involved in pollen development. Here, we demonstrated that SlPIF3 is critical for pollen development by regulating its mitotic division through auxin signalling and sugar metabolism pathways in tomato. *SlPIF3* is expressed in microsporocytes, microspores and pollen grains, but not in the tapetum (Fig. 2c), and *Slpif3‐6* exhibited a defective pollen phenotype, with poor germination rate and almost no seeds in smaller fruit (Fig. 1). Female fertility was normal in *Slpif3‐6*. Histological analysis showed that *Slpif3‐6* pollen development failed to progress through the PMI (Fig. 3c), and that its DNA duplication at PMI was completed (Fig. S4a,b), indicating that the pollen defects occur during G2/M transition or in the mitosis (M) phase of the cell cycle. In *Slpif3‐6*, the expression levels of the CDK inhibitors, *SlKRP2* and *SlKRP4*, were significantly higher than in wild‐type (Fig. S6c). Their *A. thaliana* homologues have been reported to play critical roles in the interphase of the cell cycle and to be involved in PMI during male gametogenesis (Liu *et al*., 2008). Additionally, the microtubule cytoskeleton, determined by the motor protein kinesin or myosin families, also plays a major role in meiosis and PMI (Sofroni *et al*., 2020). Many kinesin and myosin genes were expressed at lower levels in *Slpif3‐6* compared with wild‐type (Table 1). *SlPIF3* was expressed in microspores not in the tapetum (Fig. 2c), consistent with its roles at PMI. This situation is different from other bHLH family members, such as AMS and DYT1 from *A. thaliana*, TDR and EAT1 from rice and SlPIF4 from tomato, which are all involved in pollen development by regulating tapetum development (Sorensen *et al*., 2003; Zhang *et al*., 2006; Zhu *et al*., 2015; Pan *et al*., 2021).

**Fig. 8:**
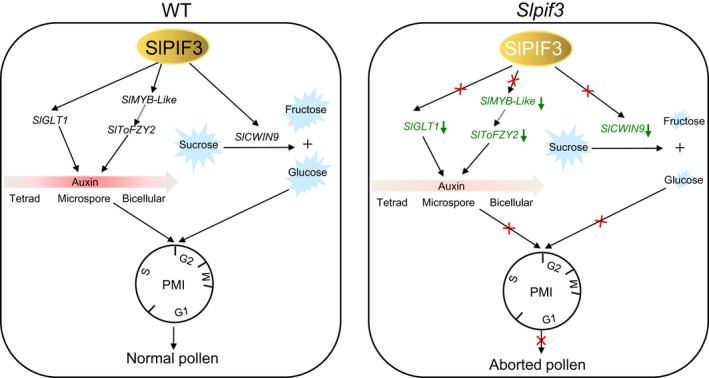
A working model for the SlPIF3‐mediated regulation of tomato pollen development. In wild‐type (WT) plants, SlPIF3 acts as a central regulatory node that integrates auxin and sugar signals to regulate pollen development of tomato. In detail, SlPIF3 positively regulates *SlMYB‐like* and *SlGLT1* to make auxin levels dynamically change during pollen development. SlPIF3 positively regulates *SlCWINV9*, which hydrolyse sucrose into fructose and glucose. Auxin and glucose act as signals to mediate the mitotic cell cycle and regulate pollen development. In *SlPIF3*‐knockout lines, the pathways for auxin and sugar are impaired, causing abnormal pollen mitosis I (PMI) and pollen development. Green arrows indicate that expression levels of the corresponding genes were downregulated. Positive effects are indicated by arrows. Dashed lines indicate interactions that have not been experimentally confirmed in this study. The big pink arrows indicate the auxin content at different stages of pollen development, a darker colour means a higher content. The red crosses indicate that these pathways are impaired.

The mitotic defect seen in *Slpif3‐6* pollen resembled the phenotype of plants with perturbations in auxin homeostasis (Feng *et al*., 2006; Sakata *et al*., 2010; Yao *et al*., 2018). Notably, the endogenous auxin content in *Slpif3‐6* anthers at the microspore stage was substantially reduced (Fig. 4d), which might be partly due to the significant downregulation of *SlToFZY2* and *SlToFZY6* expression in the anthers, as both genes are well known auxin biosynthesis genes (Fig. S4c). It has also been reported that overexpression of *GS1a* in poplar (*Populus*) significantly increased leaf glutamate and glutamine levels, which may have contributed to an increase in anthranilate synthase (*ASA1*) transcripts and elevated auxin levels in poplar leaves (Man *et al*., 2011). In our study, we found that SlPIF3 directly upregulated the expression of glutamate synthetase *SlGLT1* and that the *SlGLT1* transcript colocalised with *SlPIF3* in the microspore and was primarily expressed at the microspore stage. Knocking out *SlGLT1* significantly reduced the contents of glutamate and glutamine in anthers at the microspore stage (Fig. S9d), leading to a decrease in auxin content in *Slglt1‐2* anthers (Fig. S9d). These results further indicated that glutamine and glutamate contents may be synergistically coupled with, and contribute to, auxin biosynthesis. As expected, knocking out *SLGT1* resulted in pollen abortion and arrest at PMI, phenocopying the pollen phenotype of *Slpif3‐6* (Fig. 7) and substantiating the involvement of *SlPIF3* in the auxin pathway via its direct regulation of *SlGLT1* expression. Recently, Yao *et al*. (2018) reported that ectopic expression of *YUC2* in microsporocytes and microspores, rather than the tapetum, could rescue the fertility of *yuc2yuc6*, which failed to undergo PMI (Yao *et al*., 2018). This therefore indicated that auxin produced in microsporocytes and microspores is essential for PMI. Similarly, *SlPIF3* and *SlGLT1* were expressed in microsporocytes and microspores, consistent with their role in regulating PMI through their participation in the auxin pathway (Fig. 8). To date, although many studies have shown that auxin is essential for PMI (Cheng *et al*., 2006; Feng *et al*., 2006; Yao *et al*., 2018), the underlying molecular mechanisms are still unclear. Here, pollen viability of *Slpif3‐6* was partially rescued by exogenous auxin (Fig. 4h), accompanied by significantly lower *SlKRP2* and *SlKRP4* expression levels (Fig. S6c), suggesting that auxin is involved in PMI via its regulation of *SlKRP2* and *SlKRP4* expression. This finding is consistent with previous reports that auxin acts as a mitogenic signal to regulate the cell cycle by influencing the expression of cell cycle‐related genes (Perrot‐Rechenmann, 2010).

Invertase hydrolyses sucrose into glucose and fructose, thereby changing the hexose/sucrose ratio to maintain sugar homeostasis, which plays a fundamental role in pollen development (Zanor *et al*., 2009; Wang & Ruan, 2016). Repression of a pollen cell wall INV gene, *Nin88*, in tobacco led to reduced levels of soluble sugars transported into the pollen grains, therefore blocking PMI and causing male sterility (Goetz *et al*., 2001). Here, we found that glucose and fructose contents substantially decreased at the microspore stage of *Slpif3‐6* anthers (Fig. 4f), which may be due to the downregulation of *SlCWIN9* expression. *SlCWIN9* is directly regulated by SlPIF3 and is predominantly expressed in anthers, especially at late developmental stages (Figs 5, 6). Additionally, knocking out *SlCWIN9* significantly reduced glucose and fructose levels in anthers at the microspore stage (Fig. S9c) and led to pollen arrest at PMI (Fig. 7). Glucose, as a signalling molecule, has been reported to play prominent roles in initiating the G2/M transition in *A. thaliana* meristematic tissues (Skylar *et al*., 2011) and supplying glucose to *A*. *thaliana* suspension cells was observed to increased *CYCD2;1*, *D3;2*, *CYCA3;2* and *CYCB1;2* expression levels (Riou‐Khamlichi *et al*., 2000). Indeed, higher *SlKRP2* expression and lower *SlCYCD2;1* expression were found in *Slcwin9‐A* in microspore anthers compared with wild‐type (Fig. S9a). In addition, the *SlKinesin1* gene was expressed at lower levels in *Slwcin9‐A* (Fig. S9a). This was similar to *Slpif3‐6*, suggesting that SlPIF3 functions in pollen development by regulating the expression of *SlCWIN9* as part of anther sugar homeostasis, therefore affecting cell cycle progression and cytoskeleton development (Fig. 8). Our results indicated that both sugar and auxin act as signal molecules to regulate pollen development, in line with the increasing evidence that there is crosstalk between sugar and auxin signalling (Sagar *et al*., 2013; Min *et al*., 2014). In our study, after application of IAA, *SlVIN2* and *SlVIN9* expression in *Slpif3‐6* anthers significantly increased compared with mock‐treated anthers (Fig. S6c), indicating that auxin may also affect sugar metabolism.

Many genic male sterile (GMS) tomato mutations have been identified and their use for tomato hybrid seed production has been extensively discussed (Du *et al*., 2020). However, they have not been widely used, possibly due to the difficulty in efficiently maintaining male sterility in GMS lines compared with cytoplasmic male sterility (CMS) mutants (Chen & Liu, 2014). Although photoperiod‐thermosensitive GMS lines have been used for two‐line seed production in rice and some other crops, male sterile lines suffer from uncontrolled conversion from sterile to fertile under changing environmental conditions, and which leads to impurity of the hybrid seed. Our study demonstrated that pollen viability of the *Slpif3‐6* male sterile line can be partially restored by exogenous auxin treatment and that the male sterility trait is efficiently maintained in the next generation (Fig. 4g,h). The *Slpif3* mutant can therefore potentially be used as a female line for hybrid breeding (Fig. S10). We note that the *Slpif3* mutant pollen viability recovered by exogenous auxin application needed to be improved for commercial hybrid breeding purposes. However, this represents a highly efficient strategy to engineer a hormone‐regulated GMS system, and shows that novel male sterility lines for tomato breeding can be obtained by metabolic engineering.

## Author contributions

GL and DY conceived the study. DY, YL, LY, CP and ML generated transgenic lines and performed cytological observation and protein analysis; DY, Xiaolin Zhao and FY performed the data analysis, Xiaolin Zhao and MA helped in hormone and sugar analysis, DY, GL and Xinai Zhao wrote the article. All authors discussed and commented on the final paper.

## Supporting information


**Dataset S1** Primer sequences used in this study.

**Dataset S2** Differentially expressed genes between wild‐type and *Slpif3* mutant in tomato anther.

**Dataset S3** *Slpif3*‐III and WT‐III top down top 15 KEGG pathways in tomato anther.

**Dataset S4** Putative SlPIF3 target genes in tomato anther ChIP‐seq.

**Dataset S5** Combined ChIP‐seq and RNA‐seq data.

**Fig. S1** Production of stable *SlPIF3* transgenic tomato plants by overexpression and CRISPR/Cas9 system‐mediated gene editing.
**Fig. S2** Comparison of tomato plant morphology and flower in wild‐type, *SlPIF3*‐overexpressing lines and *Slpif3* mutant.
**Fig. S3** Knockout *SlPIF3* does not affect tomato microsporocyte meiosis, pollen wall formation and tapetum degradation.
**Fig. S4** DNA content analysis in wild‐type and *Slpif3‐6* and auxin distribution in tomato anthers.
**Fig. S5** RNA‐seq data quality analysis.
**Fig. S6** Comparison of tomato anthers genes expression levels in wild‐type, *SlPIF3*‐overexpressing lines and *Slpif3* mutant.
**Fig. S7** Genome‐wide identification of SlPIF3 binding sites.
**Fig. S8** Production of stable *SlCWIN9* and *SlGLT1* transgenic tomato plants using CRISPR/Cas9 system‐mediated gene editing.
**Fig. S9** Expression of cell cycle and auxin‐related genes and the content of auxin in wild‐type, *Slcwin9‐A* and *Slglt1‐2* tomato anthers.
**Fig. S10** The tomato hybrid seed production in a two‐line system.
**Methods S1** Generation of transgenic plants.
**Methods S2** Phenotype analysis.
**Methods S3** Transcriptome profiling and qRT‐PCR analyses.
**Methods S4** ChIP‐seq and ChIP‐qPCR analysis.
**Methods S5** Measurement of endogenous IAA and soluble sugars levels.
**Methods S6** Assay of glutamate and glutamine contents.
**Table S1** RNA‐seq reads and mapping status with tomato genome.
**Table S2** Genes involved in tapetum and pollen wall development in tomato.
Please note: Wiley Blackwell are not responsible for the content or functionality of any Supporting Information supplied by the authors. Any queries (other than missing material) should be directed to the *New Phytologist* Central Office.

## Data Availability

Sequence data from this article can be found in the NCBI Bioproject under accession code PRJNA673008. The data supporting the findings of this study are available from the corresponding author upon reasonable request.
