# Supplementary material for: Phytochrome interacting factor 3 regulates pollen mitotic division through auxin signalling and sugar metabolism pathways in tomato
Source: New Phytol. 2021 Dec 9;234(2):560–77. doi: 10.1111/nph.17878 (PMC9299586; doi:10.1111/nph.17878)
Supplement: Supplementary file 6 — Fig. S1 Production of stable SlPIF3 transgenic tomato plants by overexpression and CRISPR/Cas9 system‐mediated gene editing. Fig. S2 Comparison of tomato plant morphology and flower in wild‐type, SlPIF3‐overexpressing lines and Slpif3 mutant. Fig. S3 Knockout SlPIF3 does not affect tomato microsporocyte meiosis, pollen wall formation and tapetum degradation. Fig. S4 DNA content analysis in wild‐type and Slpif3‐6 and auxin distribution in tomato anthers. Fig. S5 RNA‐seq data quality analysis. Fig. S6 Comparison of tomato anthers genes expression levels in wild‐type, SlPIF3‐overexpressing lines and Slpif3 mutant. Fig. S7 Genome‐wide identification of SlPIF3 binding sites. Fig. S8 Production of stable SlCWIN9 and SlGLT1 transgenic tomato plants using CRISPR/Cas9 system‐mediated gene editing. Fig. S9 Expression of cell cycle and auxin‐related genes and the content of auxin in wild‐type, Slcwin9‐A and Slglt1‐2 tomato anthers. Fig. S10 The tomato hybrid seed production in a two‐line system. Methods S1 Generation of transgenic plants. Methods S2 Phenotype analysis. Methods S3 Transcriptome profiling and qRT‐PCR analyses. Methods S4 ChIP‐seq and ChIP‐qPCR analysis. Methods S5 Measurement of endogenous IAA and soluble sugars levels. Methods S6 Assay of glutamate and glutamine contents. Table S1 RNA‐seq reads and mapping status with tomato genome. Table S2 Genes involved in tapetum and pollen wall development in tomato. Please note: Wiley Blackwell are not responsible for the content or functionality of any Supporting Information supplied by the authors. Any queries (other than missing material) should be directed to the New Phytologist Central Office. [file NPH-234-560-s005.pdf]

New Phytologist

New Phytologist Supporting Information

Article title: Phytochrome Interacting Factor 3 regulates pollen mitotic division through auxin signaling and sugar metabolism pathways in tomato

Authors: Dandan Yang, Yue Liu, Muhammad Ali, Lei Ye, Changtian Pan, Mengzhuo Li, Xiaolin Zhao, Fangjie Yu, Xinai Zhao and Gang Lu

Article acceptance date: 15 November 2021

The following Supporting Information is available for this article:

Fig. S1

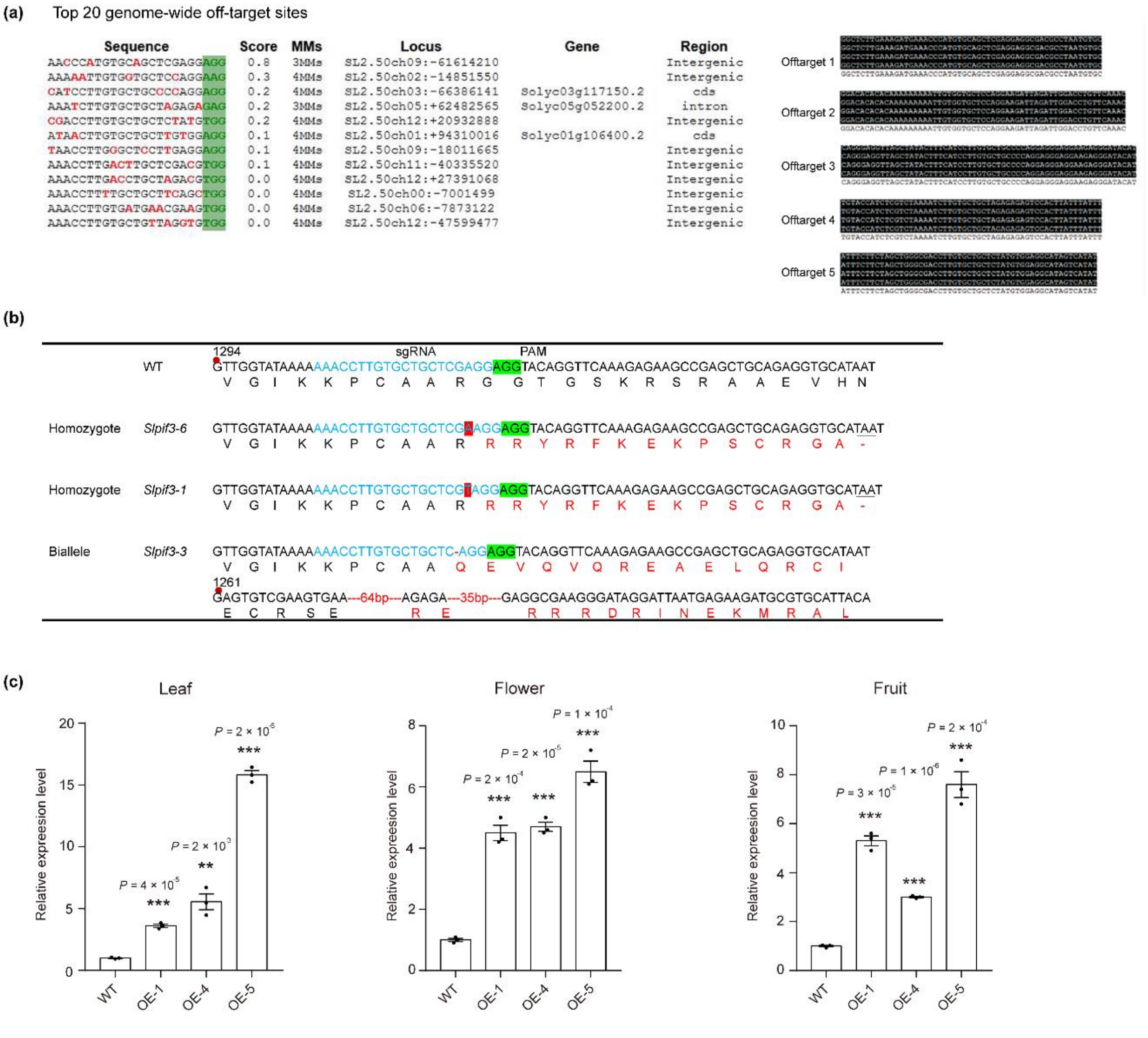

Supporting Figure S1. Production of stable *SlPIF3* transgenic tomato plants by over-expression and CRISPR/Cas9 system mediated gene editing. (a) Analysis of potential off-target sites of

SlPIF3 target sites. Potential off-target loci were predicted with the website tool CRISPR-P. No mutations were found in the putative 5 off-target sites in all tested homozygote, which were randomly selected in *Slpif3* mutant. The green shading means protospacer adjacent motifs (PAMs). (b) Schematic illustration of CRISPR/Cas9-mediated target mutagenesis of *Slpif3*. The 20 bp sgRNA adjacent to the green shading PAMs are indicated in blue. Red shading letters and red dashed lines indicate mutations. The newly created *Slpif3-6*, *Slpif3-1*and *Slpif3-3* mutants contain a 1 bp insertion of A, T and deletion of T or large fragment (red), respectively. (c) Expression level analysis of *SlPIF3* in various tissues of wild-type and over-expression transgenic tomato plant. The levels of gene expression normalized to *Ubiquitin* expression are shown relative to the wild-type level set to 1. Individual values (dots) and means (bars) are shown with three independent biological replicates, each error bar represents the mean ± SD. Asterisks indicate significant differences from wild-type. *P* values were calculated using two-tailed Student's *t* test: **, $P < 0.01$; ***, $P < 0.001$.

New Phytologist

Fig. S2

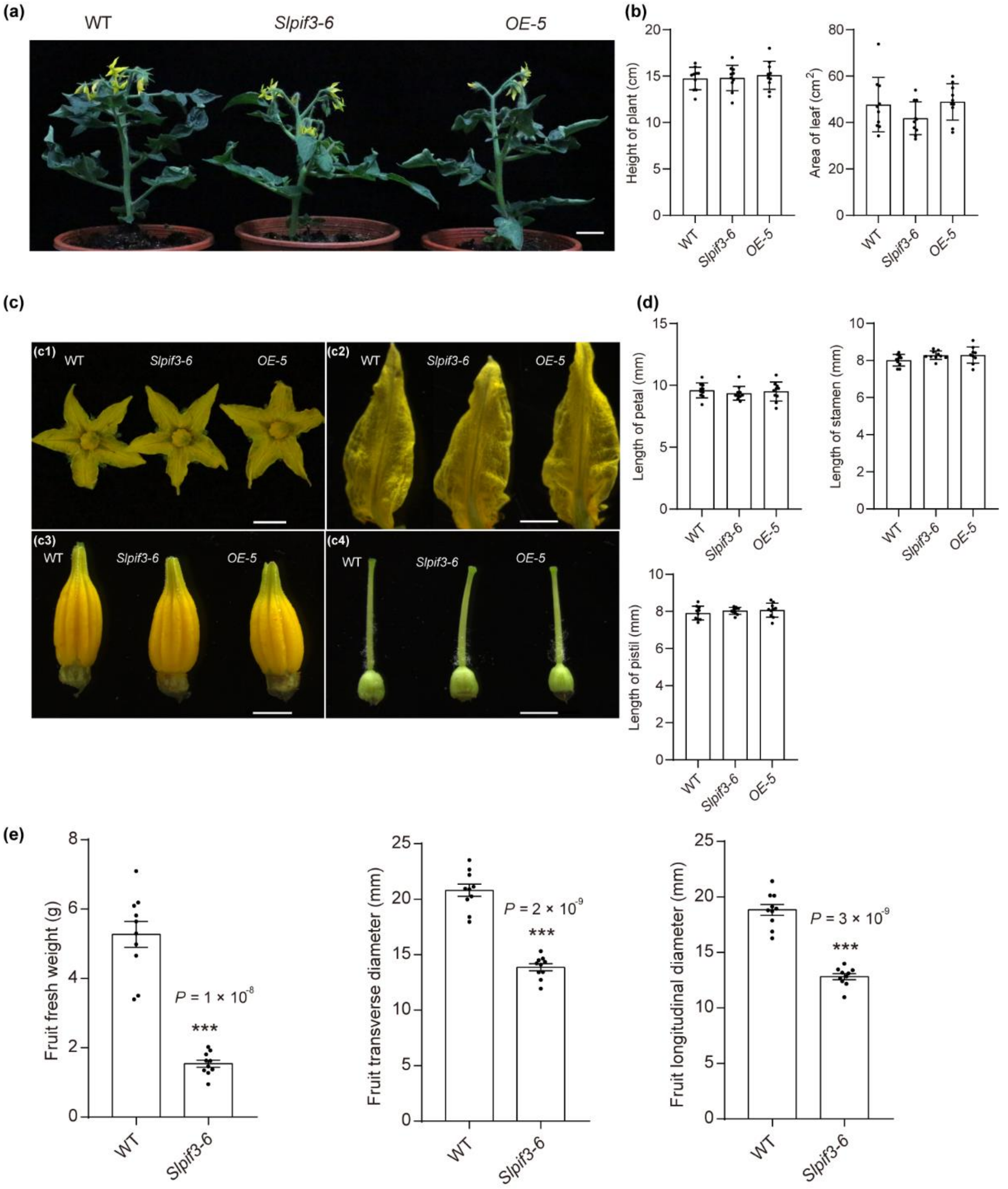

Supporting Figure S2. Comparison of tomato plant morphology and flower in wild-type, *SlPIF3*-overexpressing lines and *Slpif3* mutants. (a) The vegetative growth of wild-type, OE-5 (*SlPIF3*-overexpressing line) and *Slpif3-6*. (b) The height of plant at 40 days and leaf area of the third fully expanded leaves. (c) Mature flower buds (c1); petals (c2); stamens (c3); pistils (c4). (d) The value of length of petal, stamen and pistil. (e) Fruit fresh weight and fruit diameter of wild-type and *Slpif3-6* mutant. Each error bar represents the mean ±SE, *n* = 10 (b,d,e) biologically

independent samples. Each replicate included at least 3 plants, or 10 flowers and fruits. Asterisks indicate significant differences from wild-type control. *P* values were calculated using two-tailed Student's *t* test: ***, $P < 0.001$. Representative images in (a,b) are from one of three independent experiments with similar results. Scale bars: 1cm (a) 0.5cm (c1), 2 mm (c2-c4).

Fig. S3

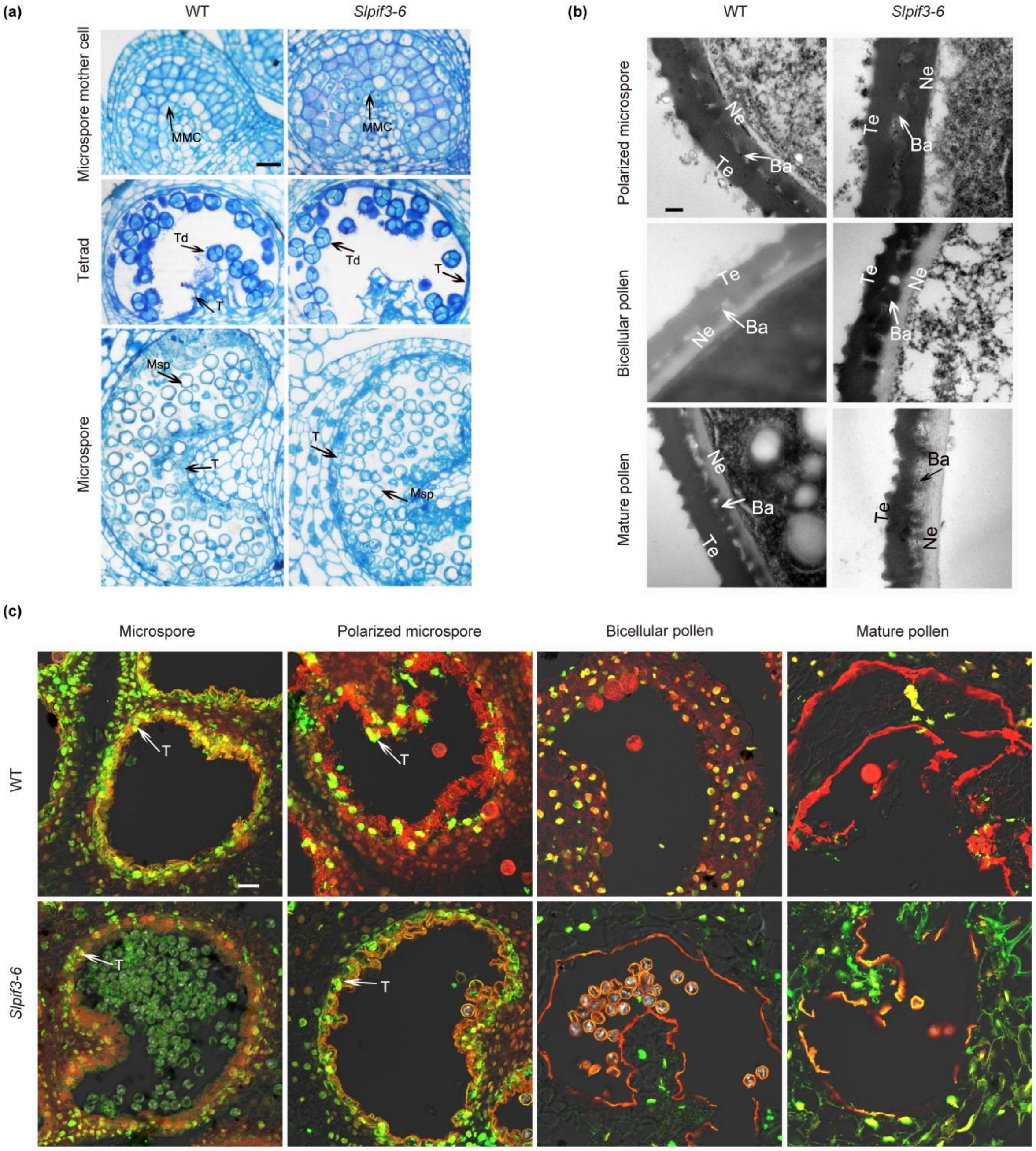

Supporting Figure S3. Knockout *SlPIF3* does not affect tomato microsporocyte meiosis, pollen wall formation and tapetum degradation. (a) Semithin cross sections of wild-type and *Slpif3* anthers at microsporocyte, tetrad, and microspore stages. There was no detectable difference between the *Slpif3-6* and wild-type anthers. (b) Close-up anthers wall of wild-type and *Slpif3* mutant from polarized microspore to mature pollen stage. (c) TUNEL (terminal deoxynucleotidyl transferase-mediated dUTP nick-end labeling) assay detected tapetum PCD in wild-type and *Slpif3-6* mutants. The green fluorescence is TUNEL positive signals and red signal is propidium iodide staining. Ba, bacula; MMC, microspore mother cell; Msp, microspore; Ne, Nexine; T, tapetum; Te, Tectum. Representative images in a,b,c are from one of three independent experiments. The similar results were detected in each experiment. Scale bar, 25 μm (a), 0.2 μm (b) and 25 μm (c).

Fig. S4

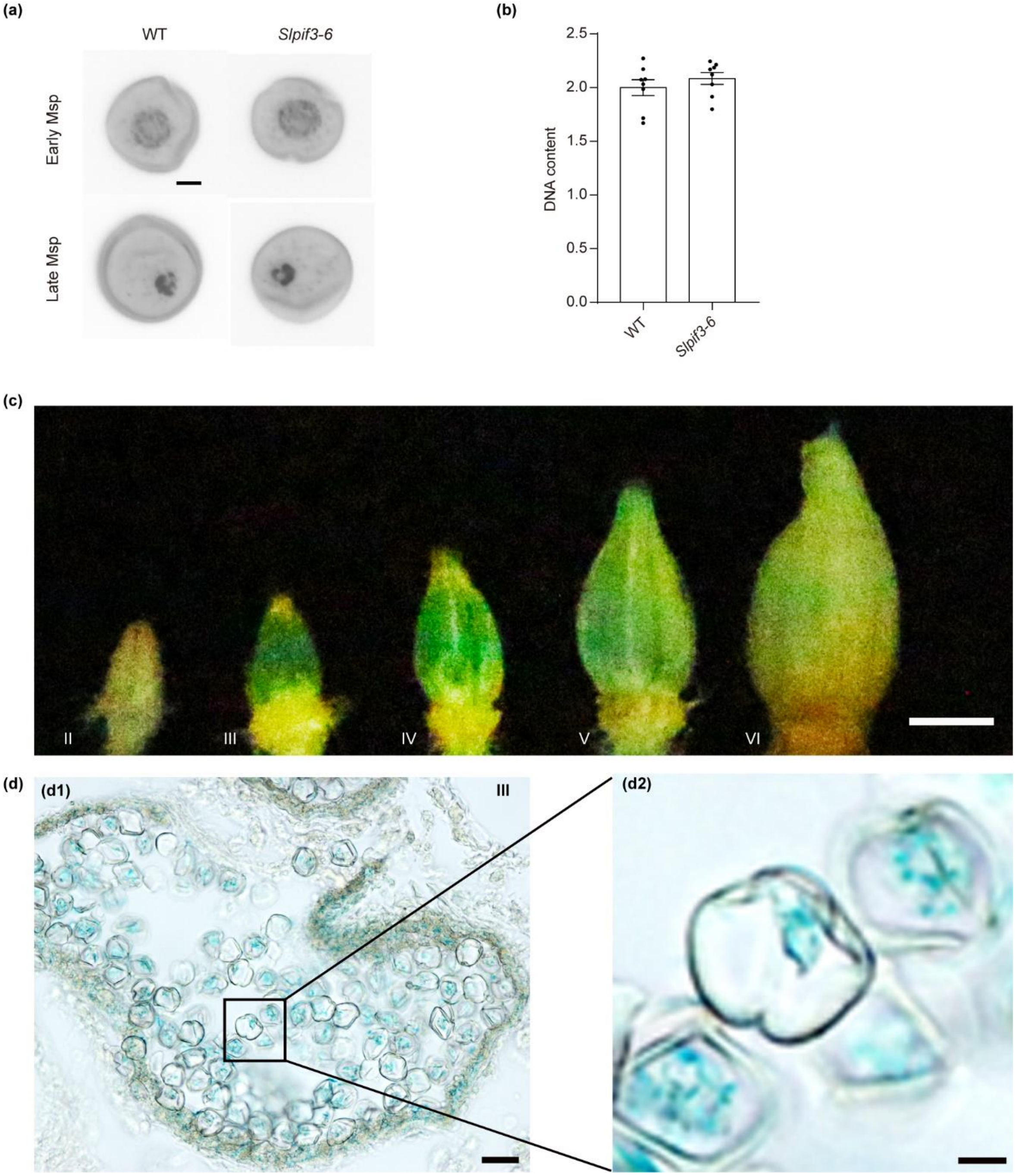

Supporting Figure S4. DNA content analysis in wild-type and *Slpif3* mutant and auxin distribution in tomato anthers. (a) Close-up of microspore of wild-type and *Slpif3-6* mutants. (b) Quantification of DAPI-stained microspore of wild-type and *Slpif3-6* mutants. The DNA content of *Slpif3-6* mutants reaches to 2C similar to wild-type. Each error bar represents the mean ±SE,

n = 8 biologically independent samples. (c) In tomato harbouring DR5-GUS, GUS (*β*-glucuronidase) signals were mainly detected in anthers from stage-III to stage-V. (d) Semithin cross sections of anthers at microspore. Scale bar, 4μm (a), 0.2 cm (c), 25 μm (d1) and 5 μm (d2).

Fig. S5

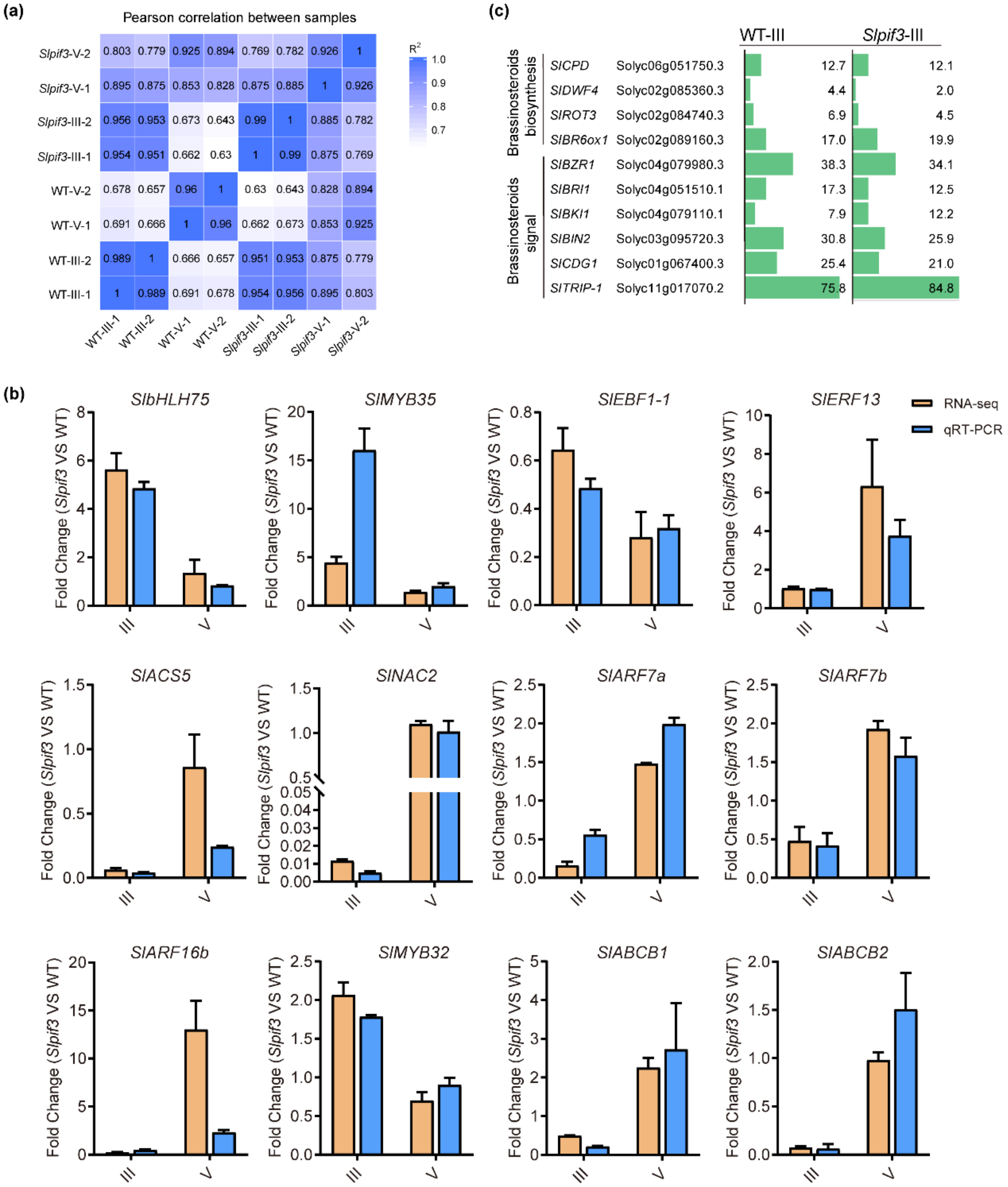

Supporting Figure S5. RNA-Seq data quality analysis. (a) Pearson correlation analysis of RNA-seq. R^2 means the square of the pearson correlation coefficient. (b) Verified RNA-seq data using quantitative real-time PCR analysis the expression levels of differentially expressed genes, which were randomly selected. For the qRT-PCR, the values and means are shown with three

independent biological replicates. Fold change in RNA-seq data was calculated based on FPKM of DEGs in *Slpif3* and wild-type at stage-III (anthers at microspore stage) and stage-V (anthers at bicellular pollen stage) in tomato, respectively; in qRT-PCR data, fold change was calculated based on the relative expression levels of DEGs in *Slpif3-6* and wild-type at stage-III (anthers at microspore stage) and stage-V (anthers at bicellular pollen stage) in tomato, respectively. Each error bar represents the mean ± SD. (c) The expression levels of brassinosteroid-related genes in wild-type and *Slpif3-6* mutant anthers based on RNA-Seq data. Values represent gene expression levels (FPKM, fragments per kilobase of exon per million fragments mapped). WT-III and *Slpif3*-III, the anthers at microspore stage in wild-type plants and *Slpif3-6* mutant, respectively.

Fig. S6

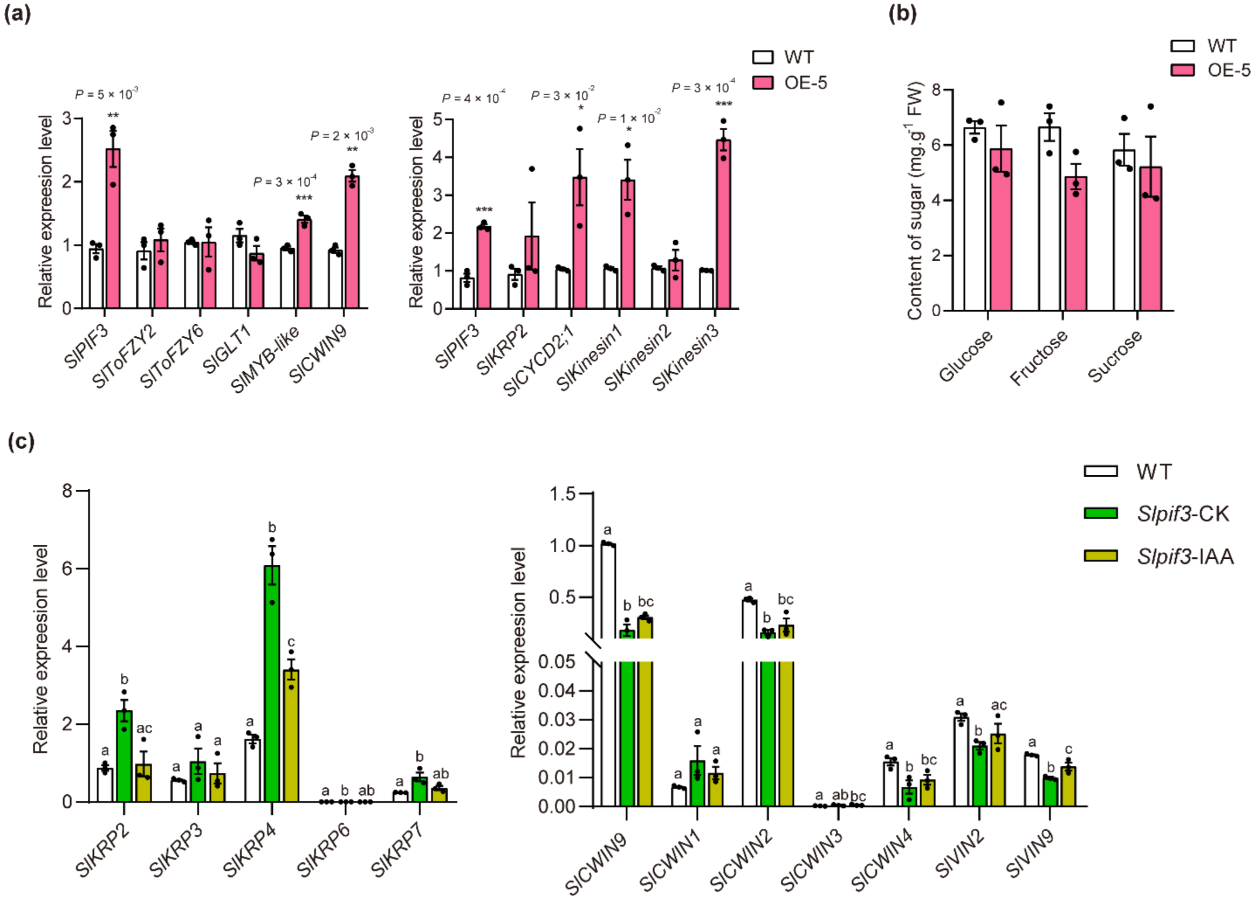

New Phytologist

Supporting Figure S6. Comparison of tomato anthers genes expression levels in wild-type, *SlPIF3*-overexpressing lines and *Slpif3* mutants. (a) qPCR analysis the expression levels of auxin-related, sugar-related and cell cycle-related genes between *SlPIF3*-overexpressing lines and wild-type anthers at microspore stage. The levels of gene expression normalized to *Ubiquitin* expression are shown relative to the wild-type level set to 1. Individual values (dots) and means (bars) are shown with three independent biological replicates, each error bar represents the mean ±SD. Asterisks indicate significant differences between *SlPIF3*-overexpressing lines and wild-type plants. P values were calculated using two-tailed Student's t test: * $P < 0.05$; **, $P < 0.01$; ***, $P < 0.001$. (b) Fructose, glucose and sucrose content in wild-type and *SlPIF3*-overexpressing lines anthers at microspore stage. Individual values (dots) and means (bars) are shown with three independent biological replicates, each error bar represents the mean ±SD. (c) qPCR analysis the expression levels of cell cycle-related and sugar-related genes between wild-type, *Slpif3* mutant treated with auxin and *Slpif3* mutant treated with mock. The levels of *SlKRP2* and *SlCWIN9* expression normalized to *Ubiquitin* expression are shown relative to the wild-type level set to 1, respectively. The values and means are shown with three independent biological replicates, each error bar represents the mean ±SD. Different letters indicate significantly different mean value at $P < 0.05$ (two-tailed Student's t test).

New Phytologist

Fig. S7

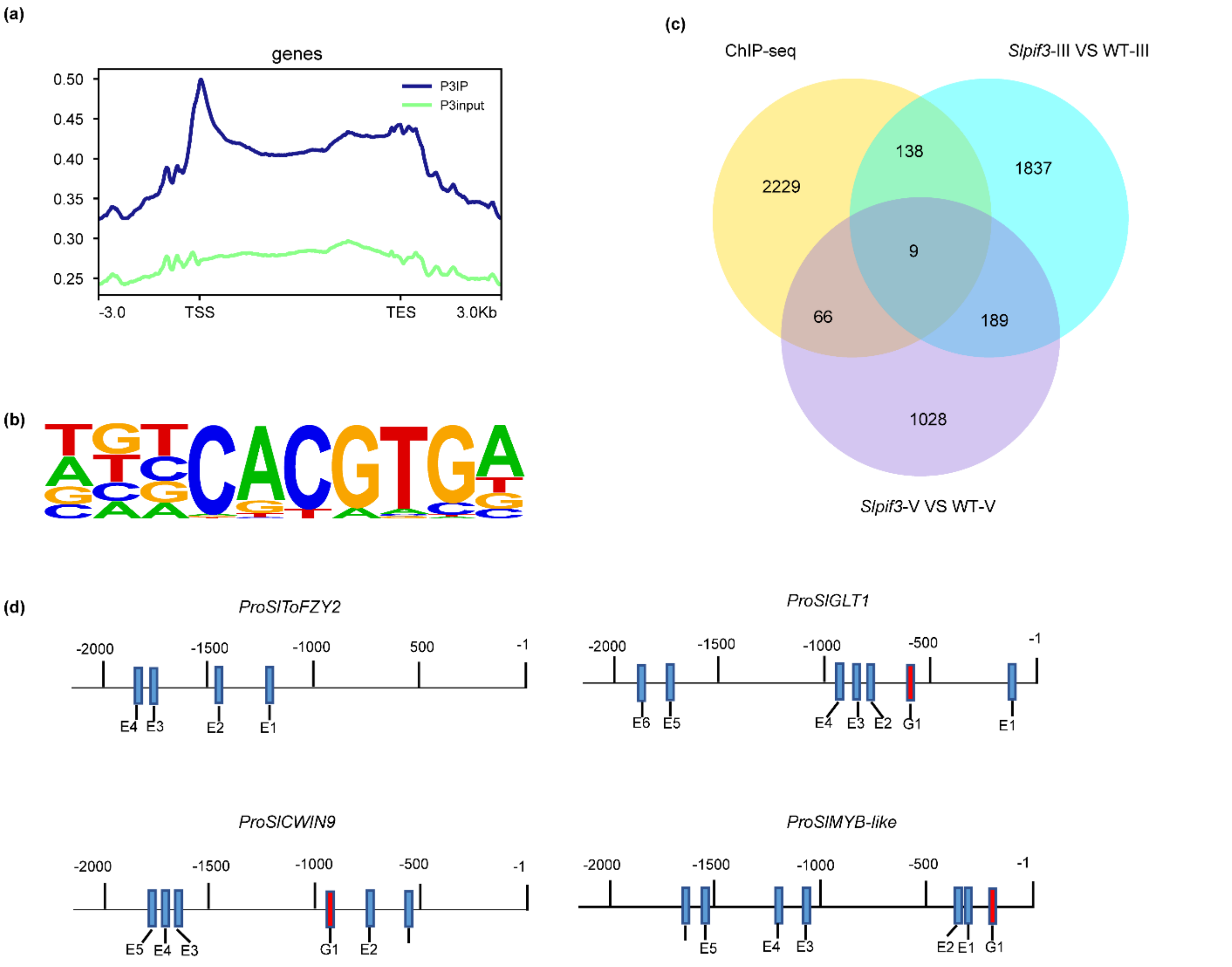

Supporting Figure S7. Genome-wide identification of SlPIF3 binding sites. (a) ChIP–seq reads distributions across gene body. TSS, transcription start site; TES, transcription end site; -3.0 kb 3000 bp upstream of the TSS of a gene; 3.0 kb, 3000bp downstream of the TES of a gene. (b) One dominant SlPIF3-binding motifs defined as a G-box (CACGTG) by Homer software. (c) Analysis of SlPIF3 target genes by combining SlPIF3 ChIP-seq data and RNA-seq data. (d) G- and E- box elements in the promoter of tomato *SlMYB-like*, *SlGLT1*, *SlCWIN9* and *SlToFZY2* gene.

Fig. S8

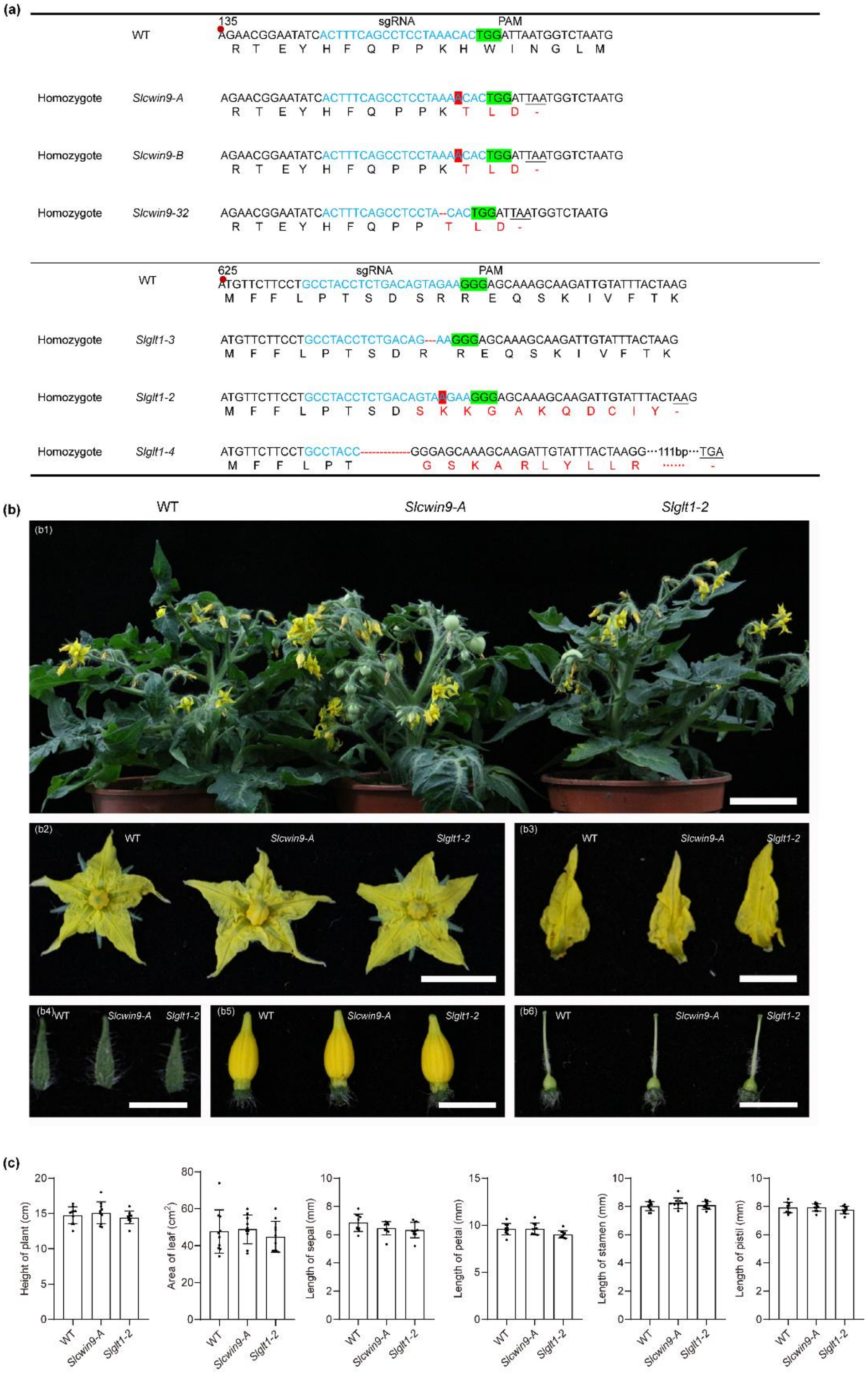
(a)
WT
Homozygote Slcwin9-A
Homozygote Slcwin9-B
Homozygote Slcwin9-32
Homozygote Slglt1-3
Homozygote Slglt1-2
Homozygote Slglt1-4
sgRNA
PAM
(b)
WT
Slcwin9-A
Slglt1-2
(c)
Height of plant (cm)
Area of leaf (cm2)
Length of sepal (mm)
Length of petal (mm)
Length of stamen (mm)
Length of pistil (mm)

Supporting Figure S8. Production of stable *SlCWINV9* and *SlGLT1* transgenic tomato plants using CRISPR/Cas9 system mediated gene editing. (a) Representative sequences of CRISPR/ Cas9-induced mutants of *SlCWIN9* and *SlGLT1*. Blue letters indicate the sequence of sgRNA and the green shading letters indicate protospacer-adjacent motif (PAM) sequences. Red shading letters and red dashed lines indicate mutations. The underlines represent stop codon. The newly created *Slcwin9-A*, *Slcwin9-B* and *Slcwin9-32* mutants contain a 1 bp insertion of A, A and 2-bases deletion, respectively. *Slglt1-3*, *Slglt1-2* and *Slglt1-4* mutants are 3-bases deletion, 1 bp insertion of A and 13-bases deletion, respectively. (b1) The vegetative growth of wild-type, *Slcwin9-A* and *Slglt1-2*; (b2) Mature flower buds; (b3) petals; (b4) sepals; (b5) stamens; (b6) pistils. (c) The height of plant at 40 days, leaf area of the third fully expanded leaves and the value of length of sepal, petal, stamen and pistil. Each error bar represents the mean ±SE, n = 10 biologically independent samples. Each replicate included at least 3 plants or 10 flowers. Scale bar, 2cm (b1), 1 cm (b2) and 0.5 cm (b3-b6).

Fig. S9

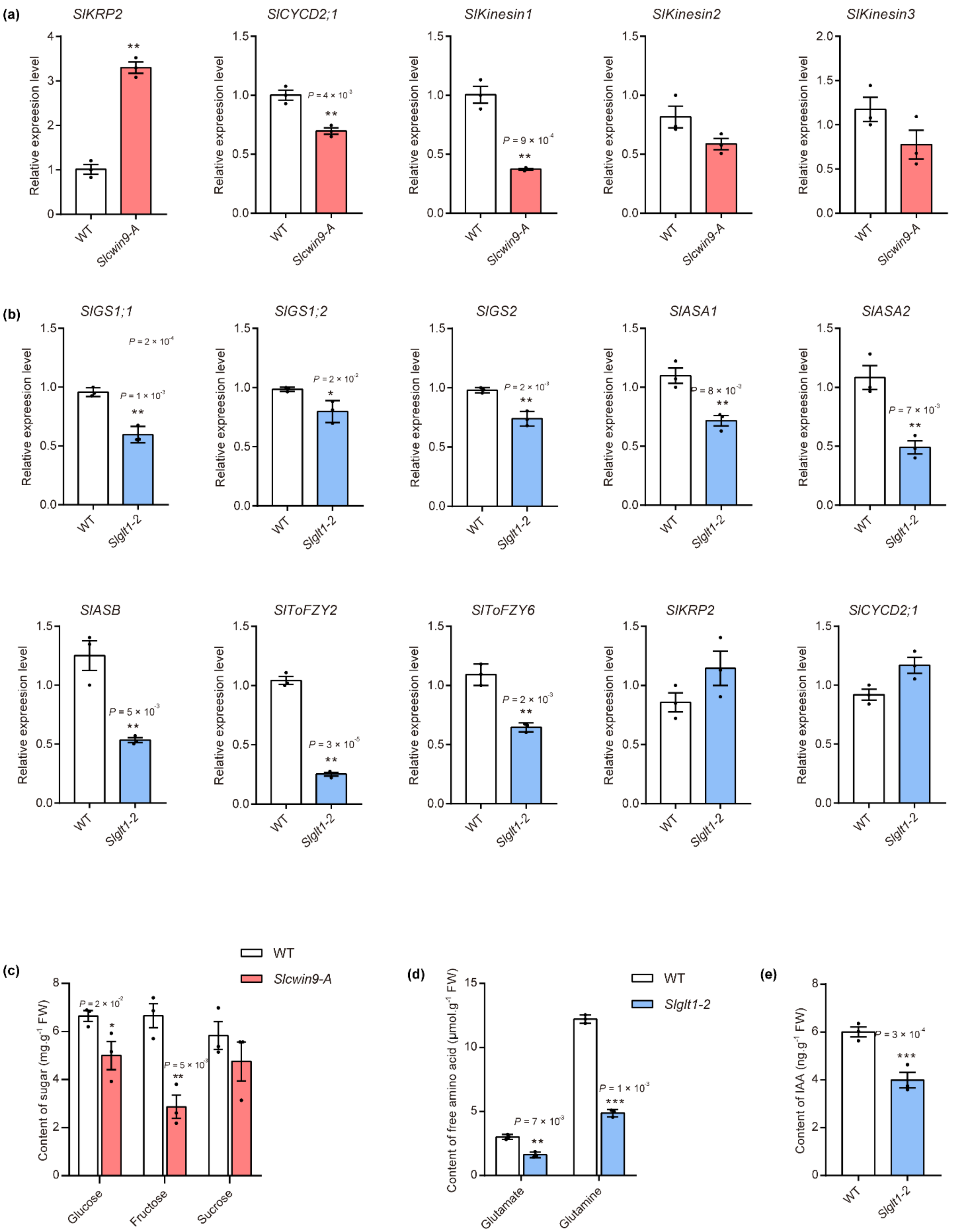
(a)
SlKRP2
SlCYCD2;1
SlKinesin1
SlKinesin2
SlKinesin3
Relative expression level
WT
Slcwin9-A
(b)
SlGS1;1
SlGS1;2
SlGS2
SlASA1
SlASA2
SlASB
SlToFZY2
SlToFZY6
SlKRP2
SlCYCD2;1
Slglt1-2
(c)
Content of sugar (mg.g-1 FW)
Glucose
Fructose
Sucrose
(d)
Content of free amino acid (μmol.g-1 FW)
Glutamate
Glutamine
(e)
Content of IAA (ng.g-1 FW)

Supporting Figure S9. Expression of cell cycle and auxin-related genes and the content of auxin in wild-type, *Slcwin9-A* and *Slglt1-2* tomato anthers. (a,b) qPCR of cell cycle and auxin-related genes in wild-type, *Slcwin9-A* and *Slglt1-2* anthers at microspore stage. The levels of genes expression normalized to *Ubiquitin* expression are shown relative to genes in wild-type level set to 1. Individual values (dots) and means (bars) are shown with three independent biological replicates, each error bar represents the mean ±SD. Asterisks indicate significant differences wild-type plants. (c) Fructose, glucose and sucrose content in wild-type and *Slcwin9-A* anthers at microspore stage. Individual values (dots) and means (bars) are shown with three independent biological replicates, each error bar represents the mean ±SD. Asterisks indicate significant differences between *Slcwin9-A* and wild-type plants. (d,e) Glutamate and glutamine content (d) and IAA content (e) in wild-type and *Slglt1-2* anthers at microspore stage. Individual values (dots) and means (bars) are shown with four independent biological replicates, each error bar represents the mean ±SD. Asterisks indicate significant differences wild-type plants. *P* values in (a,b,c,d,e) were calculated using two-tailed Student's *t* test: **, $P < 0.01$; ***, $P < 0.001$.

Fig. S10

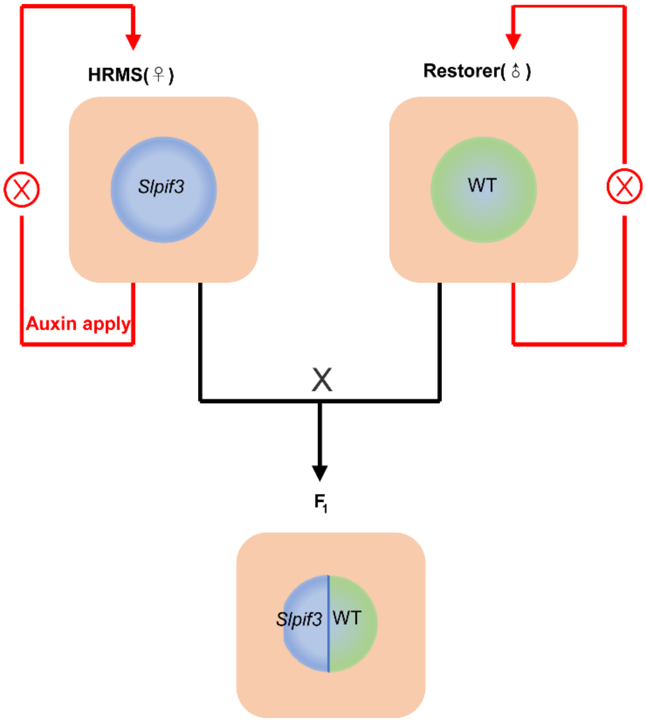

New Phytologist

Supporting Figure S10. The tomato hybrid seed production in a two-line system. The hybrid seed production in a two-line system. *Slpif3* acts as GMS and can be propagated by self-pollination (⊗) when exogenously applied auxin. HRMS: hormone regulated GMS.

Table S1 RNA-seq reads and mapping status with tomato genome

Sample name	Raw reads	Clean reads	Clean bases	Error rate	Total mapped	Multiple mapped	Uniquely mapped
WT-Ⅲ-1	54,471,670	51,990,094	7.8G	0.02%	94.67%	1.39%	93.28%
WT-Ⅲ-2	64,818,536	63,522,602	9.53G	0.02%	94.6%	1.36%	93.24%
WT-Ⅴ-1	62,963,022	60,026,290	9G	0.02%	93.05%	1.75%	91.29%
WT-Ⅴ-2	62,451,728	59,573,522	8.94G	0.02%	93.61%	1.62%	91.99%
Slpif3-Ⅲ-1	65,017,796	62,027,518	9.3G	0.02%	94.11%	1.31%	92.8%
Slpif3-Ⅲ-2	60,128,254	57,337,056	8.6G	0.02%	94.22%	1.32%	92.9%
Slpif3-Ⅴ-1	56,635,346	53,942,226	8.09G	0.02%	92.98%	2.05%	90.93%
Slpif3-Ⅴ-2	61,450,778	60,320,554	9.05G	0.02%	93.13%	1.78%	91.35%

Table S2 Genes involved in tapetum and pollen wall development in tomato (*Slpif3-6*-III VS WT-III)

Functional category	Gene name	Gene locus	Log_2FC	*P*-vale
Tapetum	*SlDYT1*	Solyc02g079810.2	-0.44005	0.030
	SlMYB80	Solyc10g005760.2	0.9043	6.49E-06
	SlMS1	Solyc04g008420.1	-0.42957	0.017
Callose	*SlQRT3*	Solyc02g068400.3	-0.39082	0.003
	SlA6	Solyc12g098560.2	0.41939	0.001
Sporopollenin	*SlCYP704B1*	Solyc01g010900.3	-0.46207	0.039
	SlTKPR1	Solyc04g008780.3	0.57764	4.74E-08
	SlTKPR2	Solyc01g068080.3	0.91148	4.74E-18
Lipid	*SlCCOAOMT1*	Solyc01g107910.3	0.71873	0.0001
	SlATCHS	Solyc09g091510.3	-0.82219	0.024

Methods S1

Generation of transgenic plants

The *SlPIF3*, *SlCWIN9* and *SlGLT1* oligonucleotide was synthesized and annealed to form single stranded RNA and was then ligated into the Bbs I sites of AtU6-sgRNA-AtUBQ-Cas9 vector for fusion to Cas9, respectively. The resulting fragments were introduced into the pCAMBIA1301 binary vector, generating pCAMBIA1301-CAS9-*SlPIF3*, pCAMBIA1301-CAS9-*SlCWIN9* and pCAMBIA1301-CAS9- *SlGLT1*. Three transgene-free homozygous mutants in the T1 generation for each mutant were tested and used in this study.

Methods S2

Phenotype analysis

Tomato flower development stages are closely correlated to flower bud size (Brukhin et al., 2003). The anthers at stage I to IV were assessed and sampled based on flower bud size and morphological characteristics, including the enclosed position of sepals and petals and the color of sepals (Peng et al., 2013; Chen et al., 2018). Alexander staining solution was made by compounding it in the following order: ethanol, 1 ml; 1% malachite green in 95% ethanol, 100 μl; distilled water, 5 ml; glycerol 2.5 ml; acid fuchsin 1% in water, 500 μl; orange G, 1% in water 50 μl; and glacial acetic acid, 400 μl. In the typical Alexander stain, aborted pollen grains are blue; nonaborted are red. The base pollen germination medium consisted of 10% (w/v) sucrose, 1.0 mmol/L $CaCl_2$, 1.0 mmol/L $Ca(NO_3)_2.4H_2O$, 1.0 mmol/L $MgSO_4$.7H2O, and 0.01% H_3BO_3. The pH of the base medium was 5.8. Pollen grains were spread uniformly on the germination medium. They were incubated for 3-5 h in dark at 28°C. After incubation, the pollen was fixed and stained with Alexander. Pollen grains were counted as germinated when the pollen tube length exceeded twice the pollen diameter. For scanning electron microscopy (SEM), pollen grains were mounted on SEM carriers and coated with gold-palladium in an Eiko Model IB5 ion coater for 10 min and vacuum desiccation. Digital images were then taken using a Hitachi Model TM-1000 scanning electron microscope (Hitachi, Japan). For transmission electron microscopy (TEM), the samples were fixed with 0.1 M phosphate-buffered saline (PBS) (pH 7.2) containing 2.5% glutaraldehyde (v/v) for 12 h at 4°C. Subsequently, they were washed thrice with 0.1 M PBS, followed by soaking in 1% osmic acid (v/v) for 1-2 h. Specimens were rewashed thrice

with 0.1 M PBS and then dehydrated through a gradient ethanol series. The specimens were embedded and polymerized in Spurr's resin and cut into 2-μm-thick sections, which were stained with 1% methylene blue. Sections were observed and photographed under a Nikon Eclipse 90i microscope (Nikon, Japan).

Methods S3

Transcriptome profiling and qRT-PCR analyses

The anthers at microspore and bicellular pollen stages from homozygous *Slpif3-6* mutant and wild-type plants were collected for RNA-seq and pooled for each of two biological replicates, each with at least 20 flowers. Total RNA was extracted from each pool using TRIZOL (Invitrogen, USA). Illumina sequencing libraries were constructed according to the manufacturer's instructions and then sequenced using an Illumina HiSeq 4000 system by Novogene Biotech (Beijing, China). Gene expression levels were calculated using the RPKM method (reads per kilobase transcriptome per million mapped reads). DEGs (differently expressed genes) were identified by adjusted *P*-value ≤ 0.05 and $\log_2$|fold change| ≥ 1. To confirm the DEGs, several randomly selected genes were analysed by qRT-PCR with the gene-specific primers listed in Dataset S4.

The tomato anther development has been classified into six different stages (I-VI) based on flower bud length (Chen *et al.*, 2018). To analyse the spatial and temporal expression of *SlPIF3*, anthers at different stages, roots, stems, leaves, and fruits were collected from 'Micro-Tom' plants. Total RNA was extracted using Total RNA Kit II (OMEGA, USA) and reverse-transcribed with PrimeScript™ RT reagent kit (Takara, Japan). The qRT-PCR was performed using SYBR® Green Realtime PCR Master Mix (Toyobo, Japan) and Bio-Rad CFX96 (Bio-Rad, USA) with triplicate for each sample. *SlUBI3* was used as an internal reference. The relative expression levels of genes were calculated using the $2^{-\Delta\Delta Ct}$ method (Kenneth & Thomas, 2002). All primers used for qRT-PCR analysis are listed in Dataset S4.

Methods S4

ChIP-seq and ChIP-qPCR analysis

The sequencing reads were aligned to tomato reference genome (SL 3.0) using the bwa program. Only uniquely mapped reads were used for further processing. MACS software was used for

peak identification in immunoprecipitation sample with the corresponding input sample as control. A strict criteria with cutoff MACS assigned false discovery rate (FDR) < 0.05 was performed for obtain high-confidence peaks (Zhang *et al.* 2008). ChIP tracks showing SlPIF3-3HA fusion protein binding sites were visualized using IGV (Helt *et al.* 2009). The peaks summit localized within 3,000-bp upstream of the TSS of a gene were classified as promoter region binding sites. Multiple EM for Motif Elicitation (MEME)-ChIP (Machanick & Bailey, 2011) was used to analysis significantly enriched motifs.
For ChIP-qPCR, the sample collection and preparation were the same as for the ChIP-Seq. The chromatin complexes containing SlPIF3-3HA fusion proteins were immunoprecipitated with an anti-HA antibody (Abcam, England), and goat anti-mouse IgG (Millipore, USA) was used as a negative control. The *Slactin2* gene was used as an internal reference. ChIP-qPCR was performed with three biological replicates with the primers listed in Supporting Information Dataset S4.

Methods S5

Measurement of endogenous IAA and soluble sugars levels

For endogenous IAA levels, fresh anthers (100 mg) were ground to a powder in liquid nitrogen with mortar and pestle. The phytohormones were extracted from the powder using 1 mL ethyl acetate which had been spiked with d2 - IAA (Sigma, Aldrich) with agitating for 10 min. The supernatant was collected after centrifugation (13,000 ***g***, 20 min, 4°C) and dried with N_2 gas. The residue was resuspended in 0.2 ml of 60% (v/v) methanol and centrifuged at 13,000 ***g*** for 10 min at 4°C. The supernatant filtered through a 0.22 μm nylon membrane was analysed by HPLC/MS-MS on an Agilent 1290 infinity HPLC system coupled with an Agilent 6460 Triple Quad LC/MS device (Agilent, USA). Four independent biological replicates were performed for each sample.

For soluble sugars levels, fresh anthers (200 mg) was ground to a powder in liquid nitrogen and homogenized in 5 mL 80% ethanol. Keeping the sample in 80°C for 30 min then centrifuged at 12,000 ***g*** for 20 min at room temperature. The supernatant was transferred for use and re-suspend pellet with 5 mL of 80% ethyl alcohol like the first time then combined with the supernatant fraction. The collected supernatant was dried by evaporation and residue was resuspended in 3 mL distilled, deionized water. After filtered through a 0.22 μm nylon membrane, the supernatant

was analysed by HPLC (Waters e2695, USA) with a 2414 RI Detector (Waters, USA) using a Waters Spherisorb NH_2 column (4.6 mm × 250 mm, 5 µm) at 35°C. The mobile phase was 80% acetonitrile at a flow rate of 1 mL/min for 22 min. The injection volume was 10 µL. Sucrose, D-(-)-fructose and D-(+)-glucose (Sigma, USA) of different gradient concentrations were used as standards. Three independent biological repeats were performed.

Methods S6

Assay of glutamate and glutamine contents

Fresh anthers (100 mg) was ground to a powder in liquid nitrogen and homogenized in 1 mL 4% sulphosalicylic acid then centrifuged at 12,000 ***g*** for 10 min at 4℃. The supernatant filtered through a 0.22 µm nylon membrane was analysed using an automated amino acid analyser Hitachi L-8900 (Hitachi, Japan) according to the manufacturer's instruction. L-Glutamate (Glu) and L-glutamine (Gln) (Sigma, USA) of different gradient concentrations were used as standards. Three independent biological repeats were performed.

References

Brukhin V, Hernould M, Gonzalez N, Chevalier C, and Mouras A. 2003**.** Flower development schedule in tomato *Lycopersicon esculentum* cv. sweet cherry. *Sex. Plant Reprod* **15**: 311–320.

Chen L, Yang D, Zhang Y, Wu L, Zhang Y, Ye L, Pan C, He Y, Huang L, Ruan YL, *et al.* 2018. Evidence for a specific and critical role of mitogen-activated protein kinase 20 in uni-to-binucleate transition of microgametogenesis in tomato. *New Phytologist* **219**: 176–194.

Heinz S, Benner C, Spann N, Bertolino E, Lin YC, Laslo P, Cheng JX, Murre C, Singh H, Glass CK. 2010. Simple combinations of lineage-determining transcription factors prime cis-regulatory elements required for macrophage and B cell identities. *Molecular Cell* **38**: 576-589.

Helt GA, Nicol JW, Erwin E, Blossom E, Blanchard SG, Chervitz SA, Harmon C, Loraine AE. 2009. Genoviz software development kit: Java tool kit for building genomics visualization applications. *BMC Bioinformatics* **10**: 266.

Kenneth JL, Thomas DS. **2002**. Analysis of relative gene expression data using real-time quantitative PCR and the 2-ΔΔCT method. *Methods* **25**: 402–408.

Machanick P, Bailey TL. 2011. MEME-ChIP: motif analysis of large DNA datasets. *Bioinformatics* **27**: 1696-1697.

Peng Z, Cheng L, He Y, Wang J, Guan X, Liu S and Lu G. 2013. Cytological study on microsporogenesis of *Solanum lycopersicum* var. Micro-Tom under high temperature stress. *Acta Ecologica Sinica* **33**: 2084-2092.

Zhang Y, Liu T, Meyer CA, Eeckhoute J, Johnson DS, Bernstein BE, Nusbaum C, Myers RM, Brown M, Li W *et al*. 2008. Model-based analysis of ChIP-Seq (MACS). *Genome Biology* **9**: R137.
